# Alcohol-associated intestinal dysbiosis impairs pulmonary host defense against *Klebsiella pneumoniae*

**DOI:** 10.1371/journal.ppat.1006426

**Published:** 2017-06-12

**Authors:** Derrick R. Samuelson, Judd E. Shellito, Vincent J. Maffei, Eric D. Tague, Shawn R. Campagna, Eugene E. Blanchard, Meng Luo, Christopher M. Taylor, Martin J. J. Ronis, Patricia E. Molina, David A. Welsh

**Affiliations:** 1 Department of Medicine, Section of Pulmonary/Critical Care & Allergy/Immunology, Louisiana State University Health Sciences Center, New Orleans, LA, United States of America; 2 Department of Microbiology, Immunology and Parasitology, Louisiana State University Health Sciences Center, New Orleans, LA, United States of America; 3 The Department of Chemistry, The University of Tennessee Knoxville, Knoxville, TN, United States of America; 4 Department of Pharmacology and Experimental Therapeutics, Louisiana State University Health Sciences Center, New Orleans, LA, United States of America; 5 Department of Physiology, Louisiana State University Health Sciences Center, New Orleans, LA, United States of America; University of Toronto, CANADA

## Abstract

Chronic alcohol consumption perturbs the normal intestinal microbial communities (dysbiosis). To investigate the relationship between alcohol-mediated dysbiosis and pulmonary host defense we developed a fecal adoptive transfer model, which allows us to investigate the impact of alcohol-induced gut dysbiosis on host immune response to an infectious challenge at a distal organ, independent of prevailing alcohol use. Male C57BL/6 mice were treated with a cocktail of antibiotics (ampicillin, gentamicin, neomycin, vancomycin, and metronidazole) via daily gavage for two weeks. A separate group of animals was fed a chronic alcohol (or isocaloric dextrose pair-fed controls) liquid diet for 10 days. Microbiota-depleted mice were recolonized with intestinal microbiota from alcohol-fed or pair-fed (control) animals. Following recolonization groups of mice were sacrificed prior to and 48 hrs. post respiratory infection with *Klebsiella pneumoniae*. *Klebsiella* lung burden, lung immunology and inflammation, as well as intestinal immunology, inflammation, and barrier damage were examined. Results showed that alcohol-associated susceptibility to *K*. *pneumoniae* is, in part, mediated by gut dysbiosis, as alcohol-naïve animals recolonized with a microbiota isolated from alcohol-fed mice had an increased respiratory burden of *K*. *pneumoniae* compared to mice recolonized with a control microbiota. The increased susceptibility in alcohol-dysbiosis recolonized animals was associated with an increase in pulmonary inflammatory cytokines, and a decrease in the number of CD4+ and CD8+ T-cells in the lung following *Klebsiella* infection but an increase in T-cell counts in the intestinal tract following *Klebsiella* infection, suggesting intestinal T-cell sequestration as a factor in impaired lung host defense. Mice recolonized with an alcohol-dysbiotic microbiota also had increased intestinal damage as measured by increased levels of serum intestinal fatty acid binding protein. Collectively, these results suggest that alterations in the intestinal immune response as a consequence of alcohol-induced dysbiosis contribute to increased host susceptibility to *Klebsiella* pneumonia.

## Author summary

Alcohol use disorders (AUD) are an established risk factor for bacterial pneumonia. Further, alcohol consumption perturbs the normal intestinal microbial communities. Intestinal microbiota dysbiosis has been linked to impaired pulmonary host defense, however the role of alcohol-associated intestinal dysbiosis on pulmonary host defense is unknown. Here, we used an innovative model system to examine the role of alcohol-mediated intestinal dysbiosis on pulmonary host defense against respiratory infection with *Klebsiella pneumoniae*, independent of direct alcohol toxicity. We found that alcohol-mediated dysbiosis significantly increases host susceptibility to *K*. *pneumoniae* infection, independent of prevailing alcohol consumption. Alcohol-dysbiosis was associated with an altered T-cell responses in both the lung and intestinal tract following *Klebsiella* infection. Alcohol-dysbiosis also lead to increased pulmonary inflammation, as well as intestinal inflammation and damage. Our study demonstrates that alcohol-mediated dysbiosis impacts host defense and contributes to the impaired host defense against pneumonia frequently seen in alcohol abusers, and thus shifts conventional understanding of mechanisms of host defense impacted by alcohol. Characterization of microbial populations and metabolic products that regulate the immune system during harmful alcohol use may reveal novel therapeutic targets, as well as host-specific microbial communities that may be central to alcohol-mediated alterations in host defense.

## Introduction

Alcohol use disorders (AUD) and respiratory infections are significant global health burdens [1, 2]. AUD are an established risk factor for bacterial pneumonia [3]. Patients with AUD are more frequently infected with highly virulent respiratory pathogens and experience increased morbidity and mortality from these infections when they occur. *Klebsiella pneumoniae* infections are overrepresented in pneumonia patients with AUD [3, 4] and AUD patients admitted to the hospital with community-acquired *Klebsiella* pneumonia experience almost double the mortality of AUD patients infected with other pathogens [4].

AUD increase the risk of pneumonia through various mechanisms, including an increased risk of aspiration of microbes from the upper alimentary tract, decreased mucus-facilitated clearance of bacterial pathogens from the upper airway, and impaired pulmonary host defenses [5]. In fact, the prevalence of oropharyngeal colonization with *K*. *pneumoniae* may be as much as four times higher in patients with AUD compared with non-AUD patients. When combined with a depressed normal gag and cough reflexes, this leads to more frequent and more severe pneumonias from Gram-negative organisms [6]. In experimental models, chronic alcohol consumption suppresses the cytokine response to infection, as well as alveolar macrophage activation and phagocytosis [7–13]. Further, chronic alcohol feeding decreases the number of circulating lymphocytes and impairs Th1 and Th17 responses to microbial challenges [14, 15]. For example, in rats infected with *K*. *pneumoniae*, alcohol suppressed the secretion of IFN-γ from Th1 cells; and augmentation of IFN-γ secretion restored pathogen clearance [14]. Moreover, acute alcohol intoxication suppresses alveolar macrophage expression of IL-23 (a cytokine which induces Th17 cells differentiation) following *Klebsiella* infection [10]. Alcohol also limits CD8+ T-cell function in the lung during influenza infection [16].

Chronic alcohol ingestion also leads to bacterial overgrowth and dysbiosis in the small and large intestine of animals and humans [17–21]. In mice, ethanol reduces the phylum *Firmicutes* [21, 22] and the genus *Lactobacillus* [20, 21], while *Enterococcus* [21, 23], *Akkermansia*, *Corynebacterium*, and *Alcaligenes* spp. increase after alcohol administration [20–22]. Beyond phylogenetic changes, chronic ethanol administration markedly reduces amino acid metabolism, and perturbs steroid, lipid, carnitine [24], and bile acid metabolism [25]. Intestinal levels of short-chain fatty acids (SCFAs), as well as saturated long-chain fatty acids (LCFAs) are lower after ethanol administration [24, 26, 27].

The gastrointestinal (GI) microbiota plays a crucial role in the immune response to bacterial and viral respiratory infections [28–31]. The GI microbiota modulates virus-specific CD4+ and CD8+ T-cells in influenza-infected mice and a population of neomycin-sensitive commensal organisms were required for a protective pulmonary immune response [28]. Similarly, the intestinal microbiota is required for optimal host defense against bacterial respiratory infections, including *K*. *pneumoniae* [32]. These reports show that the intestinal microbiota supports mucosal host defenses against pulmonary pathogens and suggest that dysbiosis will impact the integrity of the host response to infection. We hypothesized that alcohol-mediated dysbiosis increases susceptibility to *Klebsiella pneumoniae*, independent of alcohol consumption.

We found that the increased susceptibility to *Klebsiella pneumoniae* in alcohol-fed animals is, in part, mediated by gut dysbiosis, as alcohol-naïve animals recolonized with a microbiota isolated from alcohol-fed mice had an increased *K*. *pneumoniae* burden compared to mice recolonized with a control microbiota. These findings suggest that alcohol-mediated intestinal dysbiosis contributes significantly to impaired pulmonary host defense and increases susceptibility to bacterial pneumonia, a common problem in alcohol-abusing patient populations.

## Results

### Chronic alcohol model

**Fig 1:**
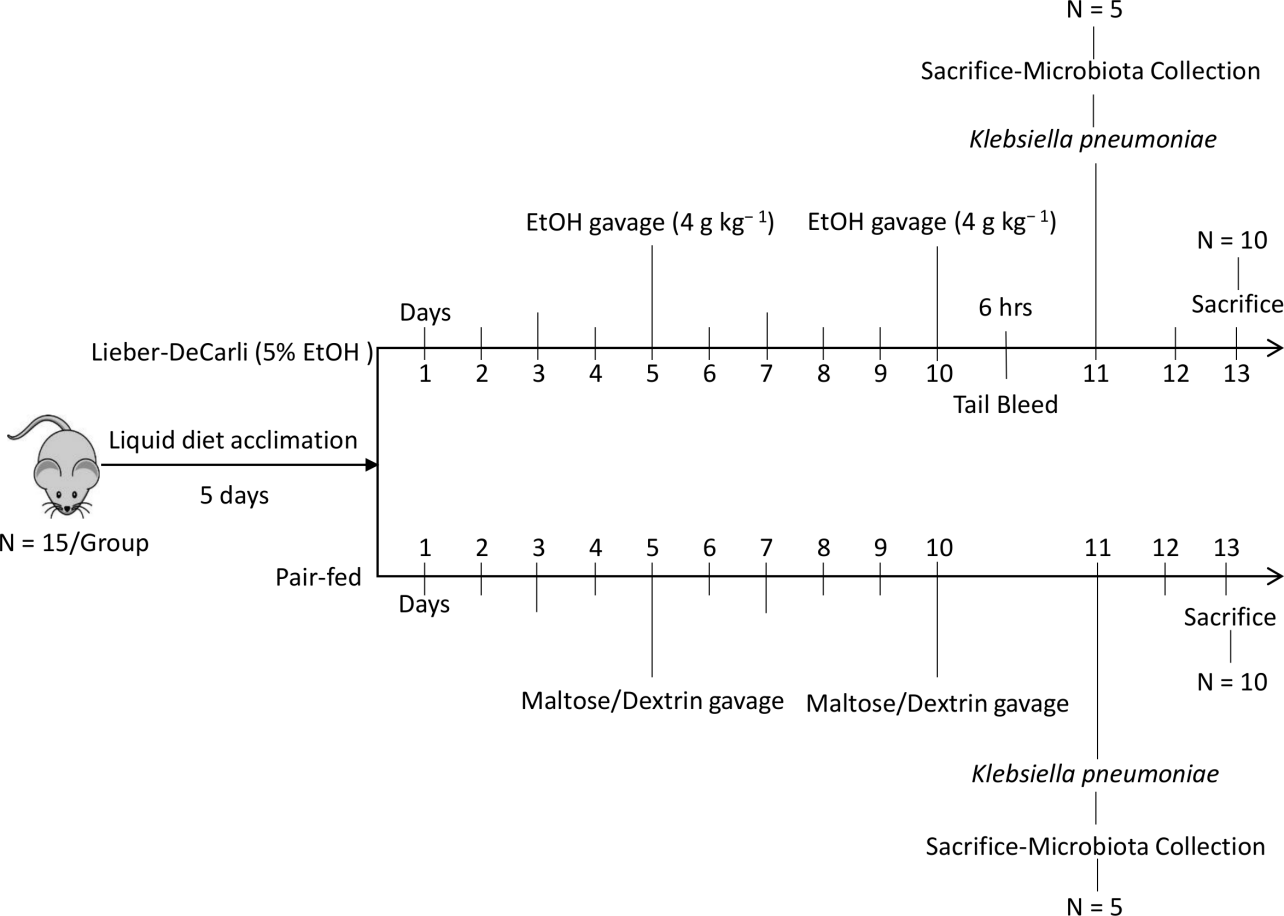
Schematic outline of the experimental protocol used in this study. C57BL/6 mice were administrered binge-on-chronic alcohol (10 days chronic and 2x binges). Following alcohol feeding groups of mice were sacrificed 24 hrs following the final binge or infected intratracheally with *K*. *pneumoniae* (~1 x 10^2^ CFU in 100 μl of PBS) and sacrificed at 48 hours post infection. 5% EtOH diet was maintained continuously throughout the experiment.

**Fig 2:**
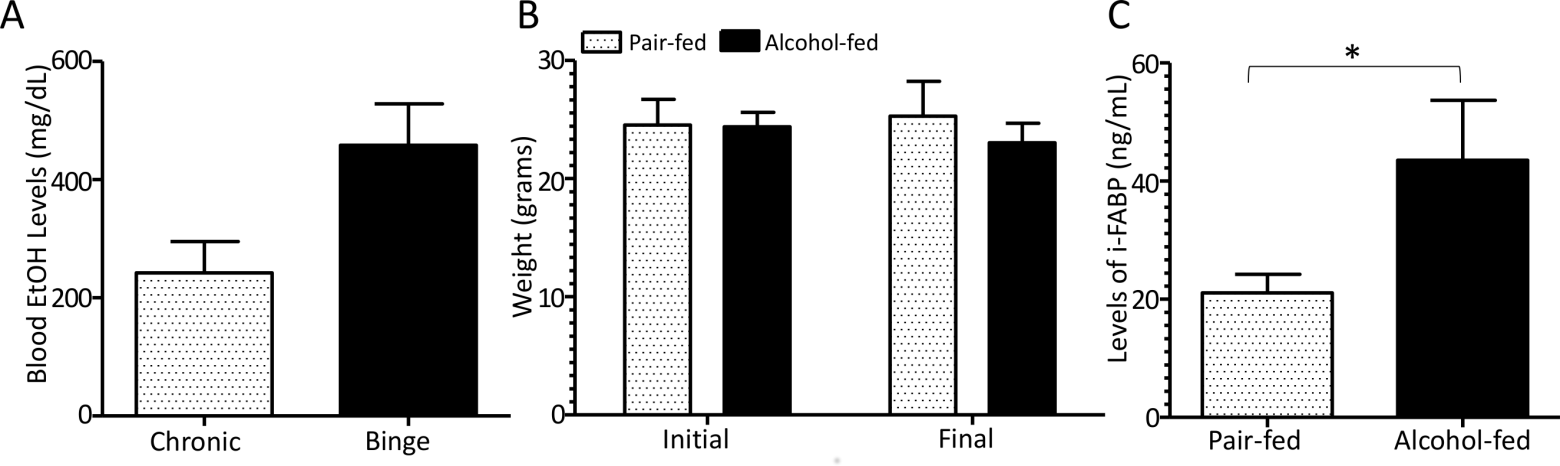
Binge-on-chronic alcohol feeding causes intestinal epithelial damage. (A) Blood alcohol levels (mg/dl) of chronic alcohol fed mice following 10 days of diet and 6 hrs following the second binge alcohol administration. (B) Body weights of pair-fed and alcohol-fed mice at baseline and post binge-on-chronic alcohol feeding (10 days chronic + 2x binge). (C) Circulating levels of intestinal fatty acid binding protien (i-FABP) in alcohol- and pair-fed control mice. Bars are the mean ± SEM, *indicates P<0.05, by Mann-Whitney U. N = 10/group.

We adapted the NIAAA chronic-binge alcohol model [33] to assess the effects of chronic alcohol feeding on host defense against *Klebsiella pneumoniae*. The experimental designed for our ***binge-on-chronic alcohol*** model is shown in Fig 1. The chronic alcohol diet produced blood alcohol concentrations of ~200 mg/dL. The binge protocol resulted in blood alcohol concentrations of ~400 mg/dL 6 hours post-binge (Fig 2A). Weight gain (Fig 2B) and daily food intake (S1 Fig) were similar in alcohol- and pair-fed mice. Binge-on-chronic alcohol consumption was also associated with a significant increase in the circulating levels of intestinal fatty acid binding protein (i-FABP), an intestinal damage biomarker, when compared to pair-fed mice (Fig 2C).

### Binge-on-chronic alcohol consumption alters the intestinal microbial community and microbial metabolic profile

**Fig 3:**
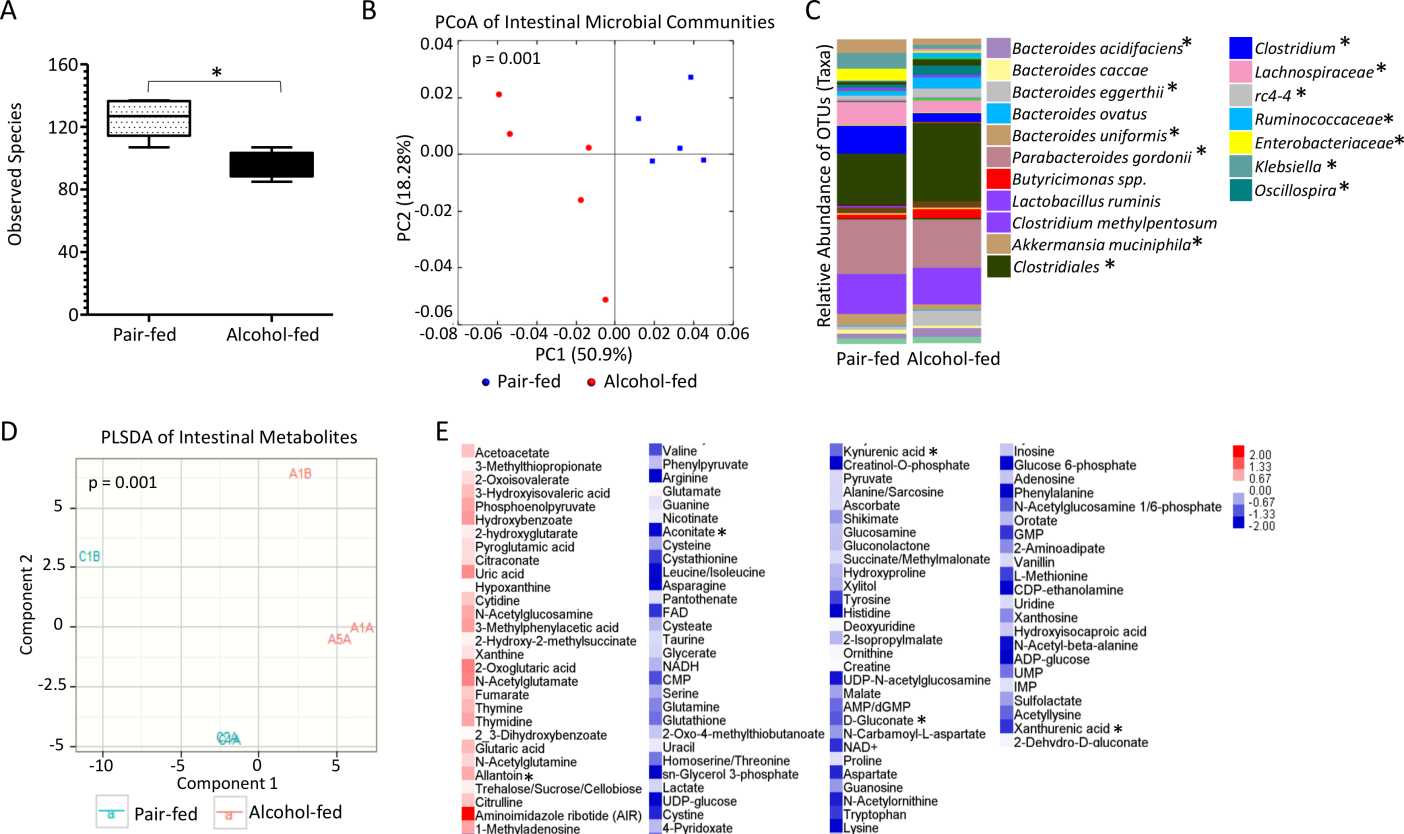
Binge-on-chronic alcohol use alters the intestinal microbial and metabolic profile. (A) Alcohol-treated mice have significantly lower alpha-diversity compared to pair-fed mice as determined by observed species via Qiime. (B) Alcohol-treated mice (red circles) showed significantly different microbial community structures from pair-fed mice (blue circles) as determined by principal coordinate analysis of the weighted UniFrac metric via Qiime. (C) Alcohol-treated mice have altered relative abundance of specific OTUs compared to pair-fed mice. (D) Alcohol-fed (red) mice had significantly different microbial metabolites from pair-fed (blue) mice, as shown by Partial Least Squares Discriminant Analysis. (E) Alcohol-treated mice have altered relative concentration of mirobial metabolites compared to pair-fed mice. *indicates P<0.05, by Mann-Whitney U with both Benjamini-Hochberg and Bonferroni corrections. N = 3-10/group.

We assessed the composition of the intestinal microbial communities in binge-on-chronic alcohol consuming mice. Binge-on-chronic alcohol feeding resulted in marked changes to the microbial alpha (α)-diversity characterized by a significant (P < 0.01) reduction in the number of observed species in alcohol-fed mice compared to pair-fed mice (Fig 3A). Beta (β)-diversity of the microbial communities from alcohol-fed and pair-fed mice was also assessed. Significant differences in the β-diversity (weighted UniFrac) microbial communities of alcohol-fed and pair-fed mice were observed as determined by principal coordinate analysis (PCoA) of the UniFrac metric via Qiime (P < 0.001) (Fig 3B). Significant differences were also observed in the unweighted UniFrac β-diversity between alcohol-fed and pair-fed mice (S2 Fig).

Comparisons between alcohol-fed and pair-fed microbial communities demonstrated significant increases in the relative abundance of specific operational taxonomic units (OTUs) in alcohol-fed mice (Fig 3C). OTUs that map to the family *Clostridiales*, the order *Ruminococcaceae*, the genus *rc4-4* and *Oscillospira*, as well as the OTUs which map to the microbial species *Bacteroides acidifaciens*, and *Bacteroides eggerthii* were increased in alcohol-fed mice. While, OTUs that map to the order *Enterobacteriaceae and Lachnospiraceae*, the genera *Clostridium and Klebsiella*, as well as the OTUs which map to the species *Bacteroides uniformis*, and *Parabacteroides gordonii*, *and Akkermansia muciniphila* were decreased in alcohol-fed mice compared to pair-fed animals (Fig 3C).

We evaluated the metabolomic profile of the microbial communities harvested from alcohol-fed and pair-fed mice. Comparisons between alcohol-fed and pair-fed mice demonstrated significant changes in the microbial metabolic profiles, as judged by Partial Least Squares Discriminant Analysis (PLSDA) (P < 0.001) (Fig 3D). Comparisons between alcohol-fed and pair-fed microbial metabolic profiles demonstrated significant increases in the relative concentration of allantoin in alcohol-fed mice compared to controls (Fig 3E). While, the relative concentration of aconitate, kynurenic acid, D-gluconate, and xanthurenic acid were significantly decreased in alcohol-fed mice compared to pair-fed animals (Fig 3E).

### Binge-on-chronic alcohol consumption increases susceptibility to *Klebsiella pneumoniae*

**Fig 4:**
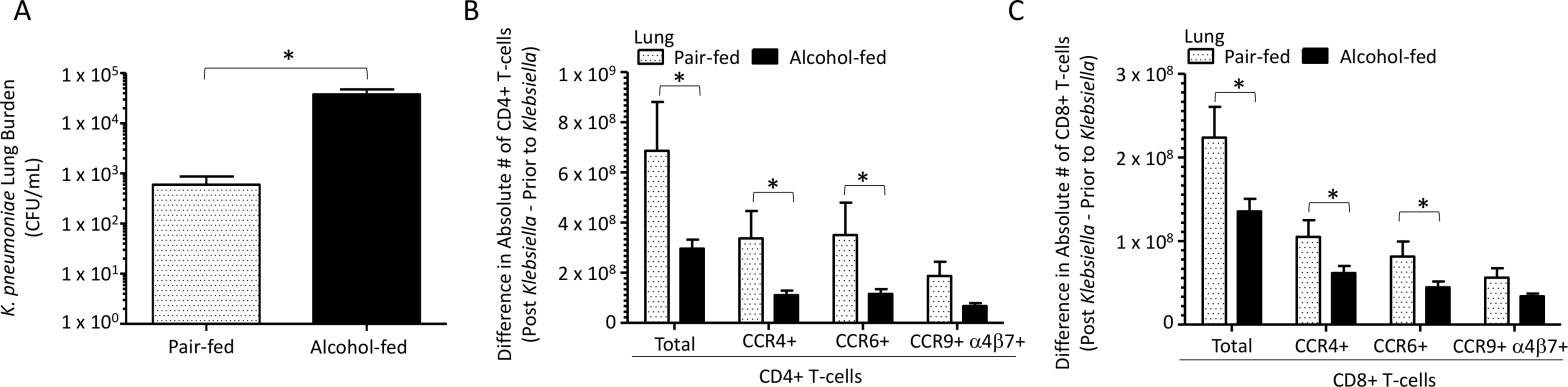
Binge-on-chronic alcohol use increases host susceptibility to *Klebsiella pneumoniae* in mice. (A) *Klebsiella* lung burden (CFU/ml) at 48 hrs. post infection in pair-fed and binge-on-chronic alcohol treated mice. (B) Difference in absolute number of homing receptor positive lung CD4+ T-cells isolated 48 hrs. post-*Klebsiella* infection from values obtained pre-infection in alcohol-fed and pair-fed mice. (C) Difference in absolute number of homing receptor positive lung CD8+ T-cells 48 hrs. post-*Klebsiella* infection from values obtained pre-infection in alcohol-fed and pair-fed mice. Bars represent the mean of the cell counts post infection minus the cell counts prior to infection ± SEM. * indicates P < 0.05, by Mann-Whitney U or by ANOVA with Dunn’s correction. N = 10/group.

We evaluated the effects of binge-on-chronic alcohol consumption on host susceptibility to *K*. *pneumoniae*. Mice receiving binge-on-chronic alcohol feeding had an increased lung burden of *K*. *pneumoniae* 48 hours post infection compared to pair-fed control mice (Fig 4A). Increased *K*. *pneumoniae* lung burden was also associated with a significant decrease in the number (expressed as the absolute number of T-cells post infection minus the absolute number of T-cells prior to infection) of CD4+ and CD8+ T-cells in the lungs of alcohol-fed mice (Fig 4B and 4C, respectively). In addition, the number of CD4+ and CD8+ T-cells that express the lung homing receptor CCR4, and the dual tropism (gut/lung) homing receptor CCR6 were decreased in the lungs of alcohol-fed mice compared to pair-fed controls (Fig 4B and 4C, respectively). In contrast, the number of lung CD4+ and CD8+ T-cells that express the gut homing receptor CCR9/a4b7 were not significantly different between pair-fed and alcohol-fed mice. We did find a significant decrease in the surface-associated markers for Th1 (CCR6^-^, CXCR3^+^, and CCR4^-^), Th2 (CCR6^-^, CXCR3^-^, and CCR4^+^), Th17 (CCR6^+^, CXCR3^-^, and CCR4^-^), central memory (CD44^+^, CD62L^+^), and effector memory (CD44^+^, CD62L^-^) CD4+ T-cells in alcohol-fed mice compared to pair-fed controls (S3A Fig). Similarly, CD45RA+ effector memory CD8+ T cell (TEMRA) counts were decreased in the lungs of alcohol-fed mice compared to pair-fed controls (S3B Fig). While the levels of both central memory and effector memory CD8+ T-cells were similar between alcohol- and pair-fed mice (S3B Fig).

**Fig 5:**
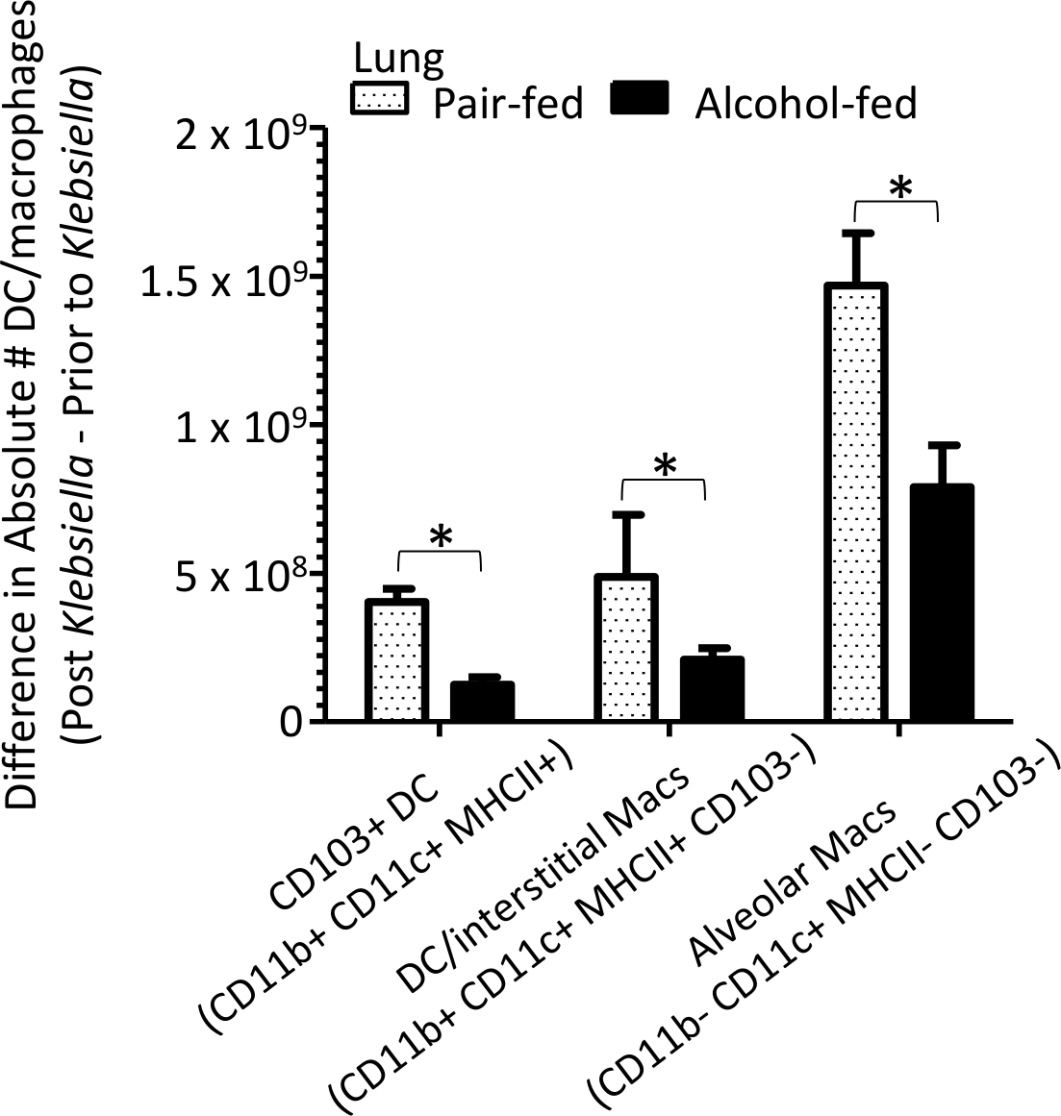
Binge-on-chronic alcohol use alters the levels of pulmonary macrophages and dendritic cells. Difference in absolute number of CD103+ dendritic cells, DC/interstitial macrophages, and aveolar macrophages isolated 48 hrs. post-*Klebsiella* infection from values obtained pre-infection in alcohol-fed and pair-fed mice. Bars represent the mean of the cell counts post infection minus the cell counts prior to infection ± SEM, * indicates P < 0.05, by ANOVA with Dunn’s correction. N = 10/group.

**Fig 6:**
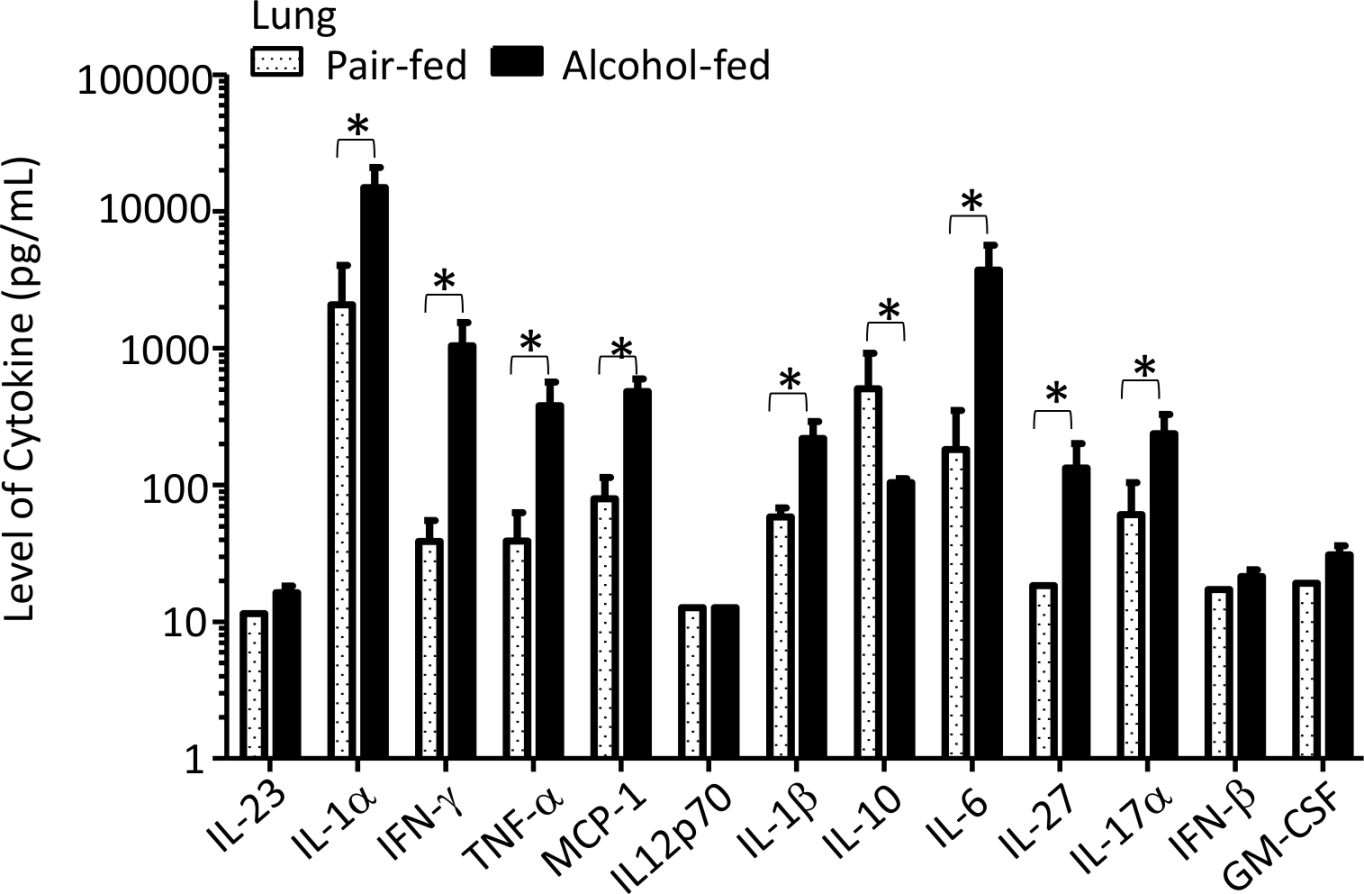
Binge-on-chronic alcohol use alters acute pulmonary inflammatory cytokines. Levels of pulmonary acute inflammatory cytokines were asssesed 48 hrs. post-*Klebsiella* infection via LEGENDplex 13-ples assay kit. Bars represent the mean cytokine level ± SEM, * indicates P < 0.05, by ANOVA with Dunn’s correction. N = 10/group.

We then investigated pulmonary macrophage and dendritic cell populations, as well as acute inflammatory cytokines, following *K*. *pneumoniae* infection. CD103+ dendritic cells (CD11b^+^, CD11c^+^, MHCII^+^, CD103^+^), DC/interstitial macrophages (CD11b^+^, CD11c^+^, MHCII^+^, CD103^-^), and alveolar macrophages (CD11b^-^, CD11c^+^, MHCII^-^, CD103^-^) were significantly different between pair-fed and alcohol-fed mice following *K*. *pneumoniae* (Fig 5). We also evaluated the levels of pulmonary acute cytokines between alcohol- and pair-fed mice following *K*. *pneumoniae* infection. We found that the levels of IL-1α, INF-γ, TNF-α, MCP-1, IL-1β, IL-10, IL-6, IL-27, and IL-17α in the lungs post infection were significantly different between alcohol- and pair-fed mice (Fig 6). Conversely the levels of IL-23, IL12p70, IFN-β, and GM-CSF were not significantly different between alcohol- and pair-fed mice (Fig 6).

We then examined the requirement for CD4+ and CD8+ T-cells in optimal host defense against respiratory infection with *K*. *pneumoniae*, as T-cells are not primarily thought to play a role in host defense against bacterial pathogens. However, our results show that monoclonal antibody mediated depletion of CD4+ or CD8+ T-cells resulted in a significant increase in the burden of *K*. *pneumoniae* in the lungs of mice when compared to isotype (control) treated mice (S4A Fig). Depletion of both CD4+ and CD8+ T-cells in the lungs prior to respiratory infection was confirmed via flow cytometry (S4B Fig).

### Fecal adoptive transfer model description

**Fig 7:**
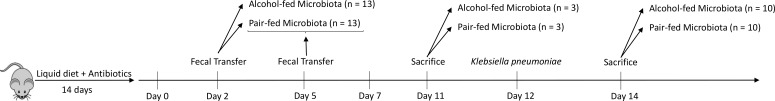
Schematic outline of the experimental protocol used in this study. C57BL/6 mice were administrered antibiotics daily for 14 days. Following antibiotic treatment mice were recolonized with cecal microbiota from alcohol-fed or pair-fed mice. Groups of mice were sacraficed 7 days following the final recolonization or infected intratracheally with *K*. *pnuemoniae* (~1x10^2^ CFU in 100 μl of PBS) and sacrificed at 48 hours post infection.

**Fig 8:**
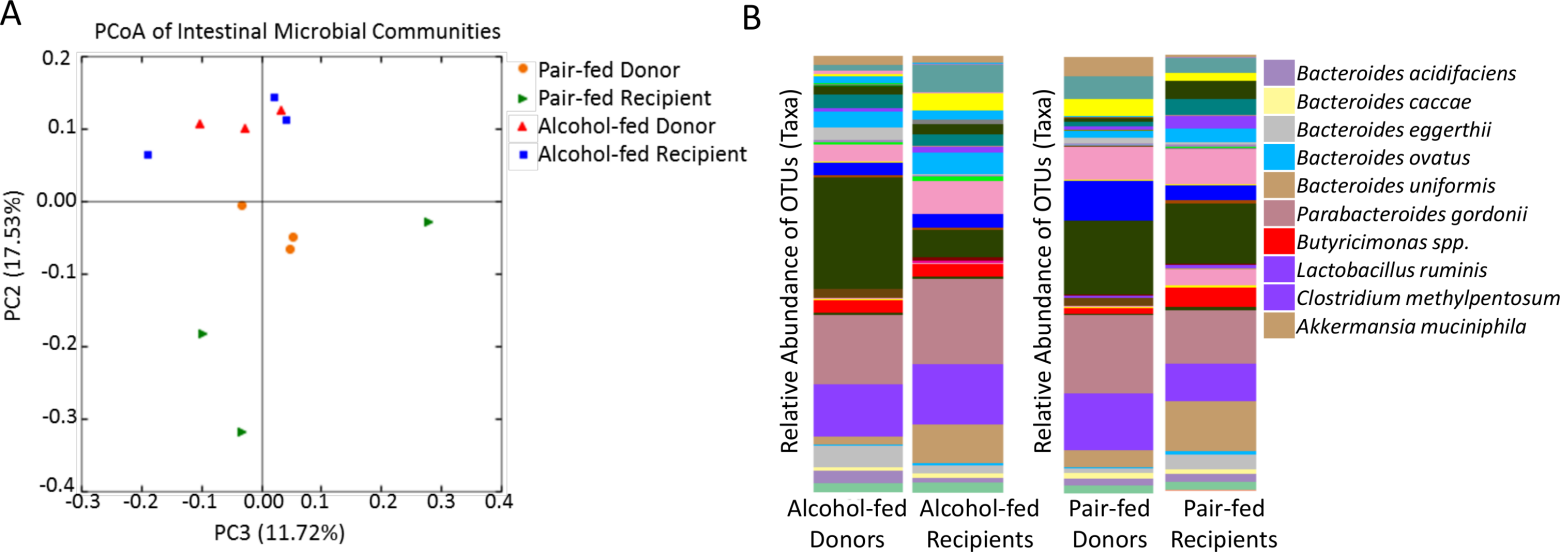
Adoptive transfer maintains microbial community structure. (A) The microbial communities of alcohol-dysbiosis recolonized mice (blue squares) compared to the microbial communities of alcohol-fed donor mice (red triangles), and the microbial communities of pair-fed recolonized mice (green triangles) compared to the microbial communities of pair-fed donor mice (orange circles). (B) Relative abundance of OTUs are similar between alcohol-microbiota recipient and alcohol-microbiota donor animals, as well as pair-fed microbiota recipient and pair-fed microbiota donor animals.

We developed a fecal adoptive transfer model to investigate the effects of alcohol-induced dysbiosis on host defense and to isolate these from the direct effects produced by alcohol on cells and tissues. The experimental designed for the fecal adoptive transfer model is shown in Fig 7. We first assessed the composition of the intestinal microbial communities between the microbiota from alcohol-fed donor animals compared to the microbiota harvested from mice recolonized with an alcohol-dysbiosis, as well as the microbiota from pair-fed donor animals compared to the microbiota harvested from mice recolonized with microbiota from pair-fed animals. Microbiota composition comparisons were assessed following one week of recolonization. Analysis of the β-diversity (weighted UniFrac) metric demonstrated the microbial communities were different between the alcohol-microbiota donor and alcohol-microbiota recipient mice (P = 0.02), as well as a different between the pair-fed microbiota donor and pair-fed microbiota recipient mice (P = 0.02) (Fig 8A). In addition, several changes in the relative abundance of specific OTUs in alcohol-microbiota donor and alcohol-microbiota recipient mice and the pair-fed microbiota donor and pair-fed microbiota recipient mice were observed (Fig 8B). The distinction between alcohol-microbiota and pair-fed microbiota in the recolonized mice was preserved and strongly significantly different (P = 0.0001).

**Fig 9:**
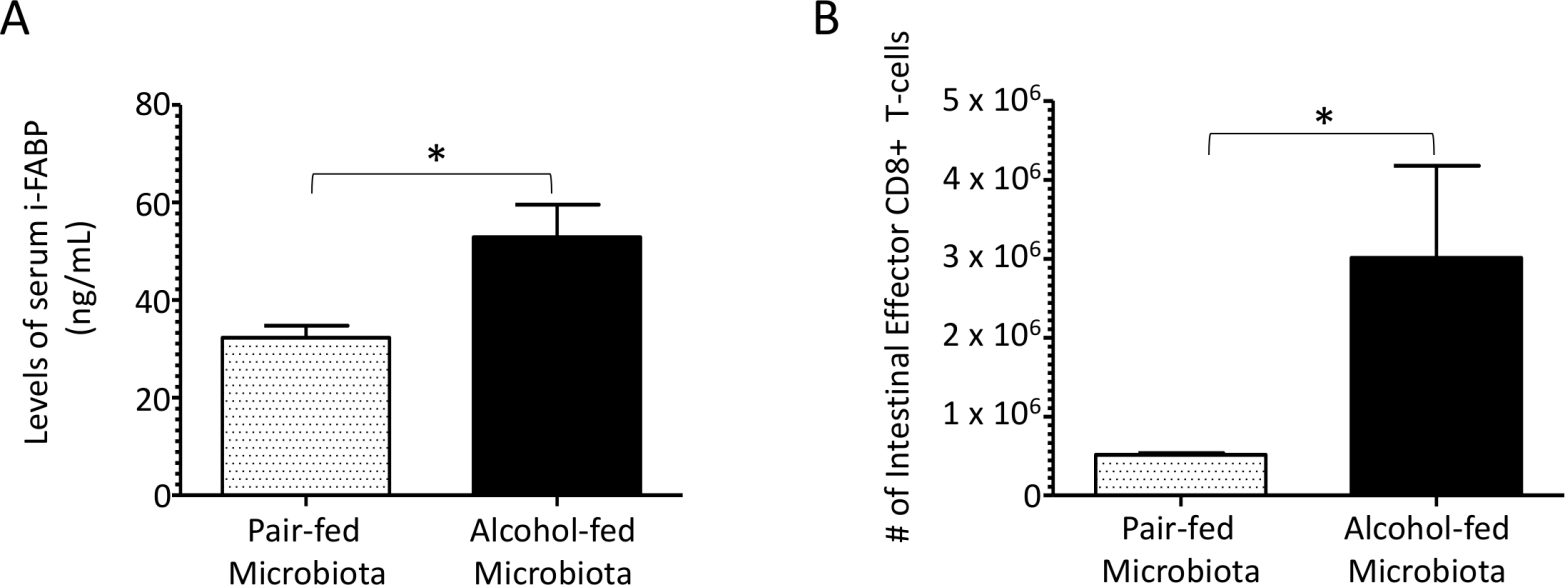
Alcohol-dysbiosis significantly increases intestinal barrier damage and inflammation. (A) Circulating levels of intestinal fatty acid binding protien (i-FABP) in alcohol-dysbiosis and pair-fed recolonized mice. (B) Absolute number of effector (CD44+, CD62L-) CD8+ T-cells in the intestine of alcohol-dysbiosis and pair-fed recolonized mice. Bars are the mean ± SEM, *indicates P<0.05, by Mann-Whitney U. N = 10/group.

We next evaluated intestinal inflammation following microbial recolonization, as the intestinal tract was affected by binge-on-chronic alcohol consumption. Our results show that mice recolonized with an alcohol-dysbiotic microbiota had a significant increase in the circulating levels i-FABP (Fig 9A) and an increase in the absolute number of intestinal effector CD8+ T-cells (CD8^+^, CD44^+^, and CD62L^-^), a marker of intestinal inflammation, when compared to pair-fed mice (Fig 9B).

### Alcohol-dysbiosis increases susceptibility to *Klebsiella pneumoniae* independent of alcohol consumption

**Fig 10:**
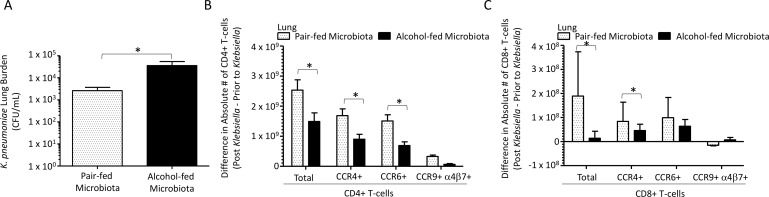
Alcohol-associated dysbiosis increases host susceptibility to *Klebsiella pneumoniae* in mice. (A) *Klebsiella* lung burden (CFU/ml) at 48 hrs. post infection in pair-fed recolonized and alcohol-dysbiosis recolonized treated mice. (B) Difference in absolute number of homing receptor positive lung CD4+ T-cells isolated 48 hrs. post-*Klebsiella* infection from values obtained pre-infection in alcohol-dysbiosis and pair-fed recolonized mice. (C) Difference in absolute number of homing receptor positive lung CD8+ T-cells 48 hrs. post-*Klebsiella* infection from values obtained pre-infection in alcohol-dysbiosis and pair-fed recolonized mice. Bars represent the mean of the cell counts post infection minus the cell counts prior to infection ± SEM. * indicates P < 0.05, by Mann-Whitney U or by ANOVA with Dunn’s correction. N = 10/group.

We examined the effects of alcohol-dysbiotic microbiota recolonization on host susceptibility to *K*. *pneumoniae*. Mice recolonized with an alcohol-dysbiotic microbiota had an increased lung burden of *K*. *pneumoniae* 48 hours post infection compared to mice recolonized with a pair-fed microbiota (Fig 10A). Increased *K*. *pneumoniae* lung burden was also associated with a decrease in the number of CD4+ and CD8+ T-cells in the lungs of alcohol-microbiota recolonized mice (Fig 10B and 10C, respectively). In addition, the number of CD4+ and CD8+ T-cells that express the lung homing receptor CCR4, and the dual tropism (gut/lung) homing receptor CCR6 were significantly decreased in the lungs of mice recolonized with microbiota from alcohol-fed mice compared to pair-fed microbiota recolonized animals (Fig 10B and 10C, respectively). A decrease in the surface associated markers for Th1 and central memory CD4+ T-cells were observed in mice recolonized with microbiota from alcohol-fed mice (S5A Fig). Similarly, the levels of both central memory and effector memory CD8+ T-cells were significantly decreased in mice recolonized with microbiota from alcohol-fed mice compared to pair-fed microbiota recolonized animals (S5B Fig).

**Fig 11:**
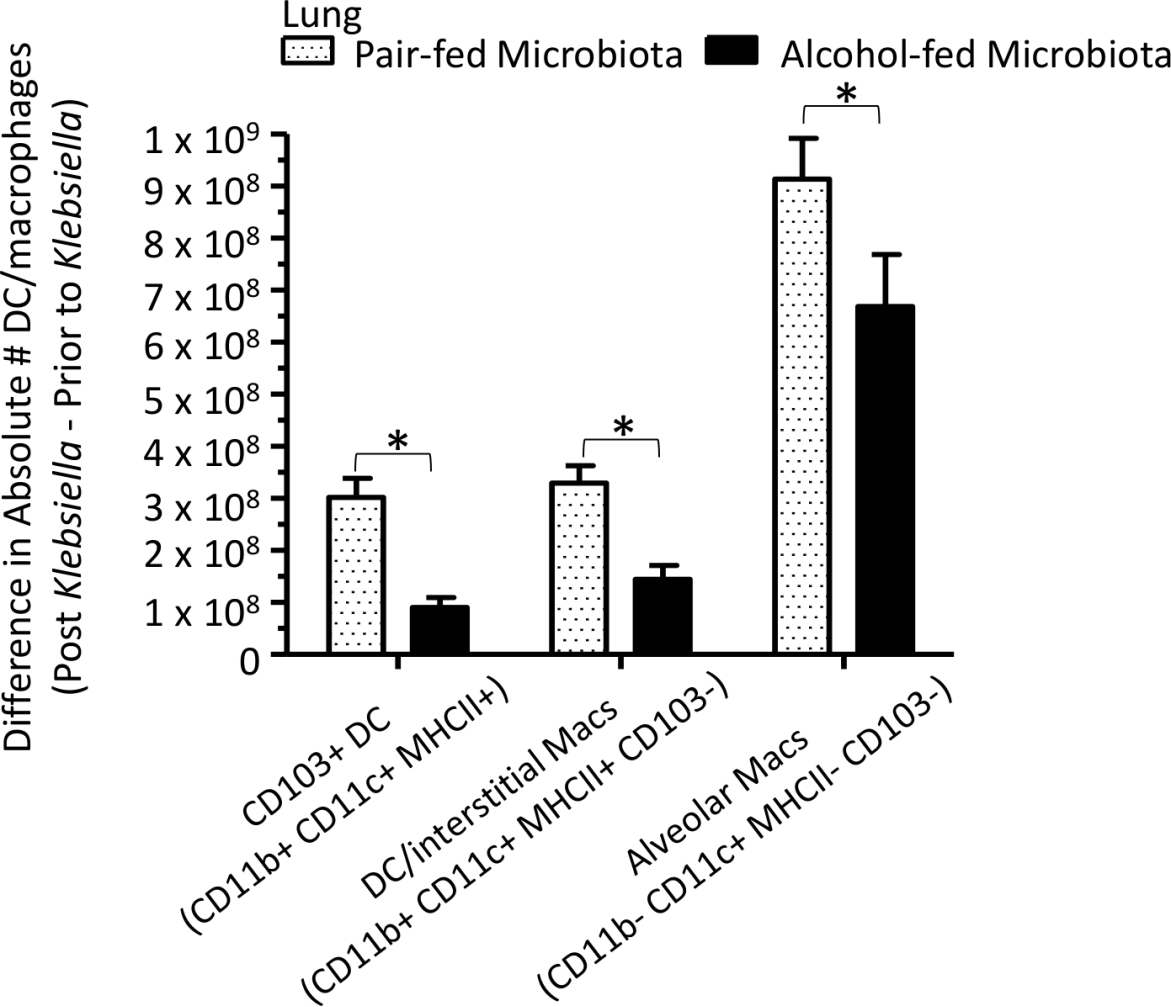
Alcohol-associated dysbiosis alters the levels of pulmonary macrophages and dendritic cells. Difference in absolute number of CD103+ dendritic cells, DC/interstitial macrophages, and aveolar macrophages isolated 48 hrs. post-*Klebsiella* infection from values obtained pre-infection in alcohol-dysbiosis and pair-fed recolonized mice. Bars represent the mean of the cell counts post infection minus the cell counts prior to infection ± SEM, * indicates P < 0.05, by ANOVA with Dunn’s correction. N = 10/group.

**Fig 12:**
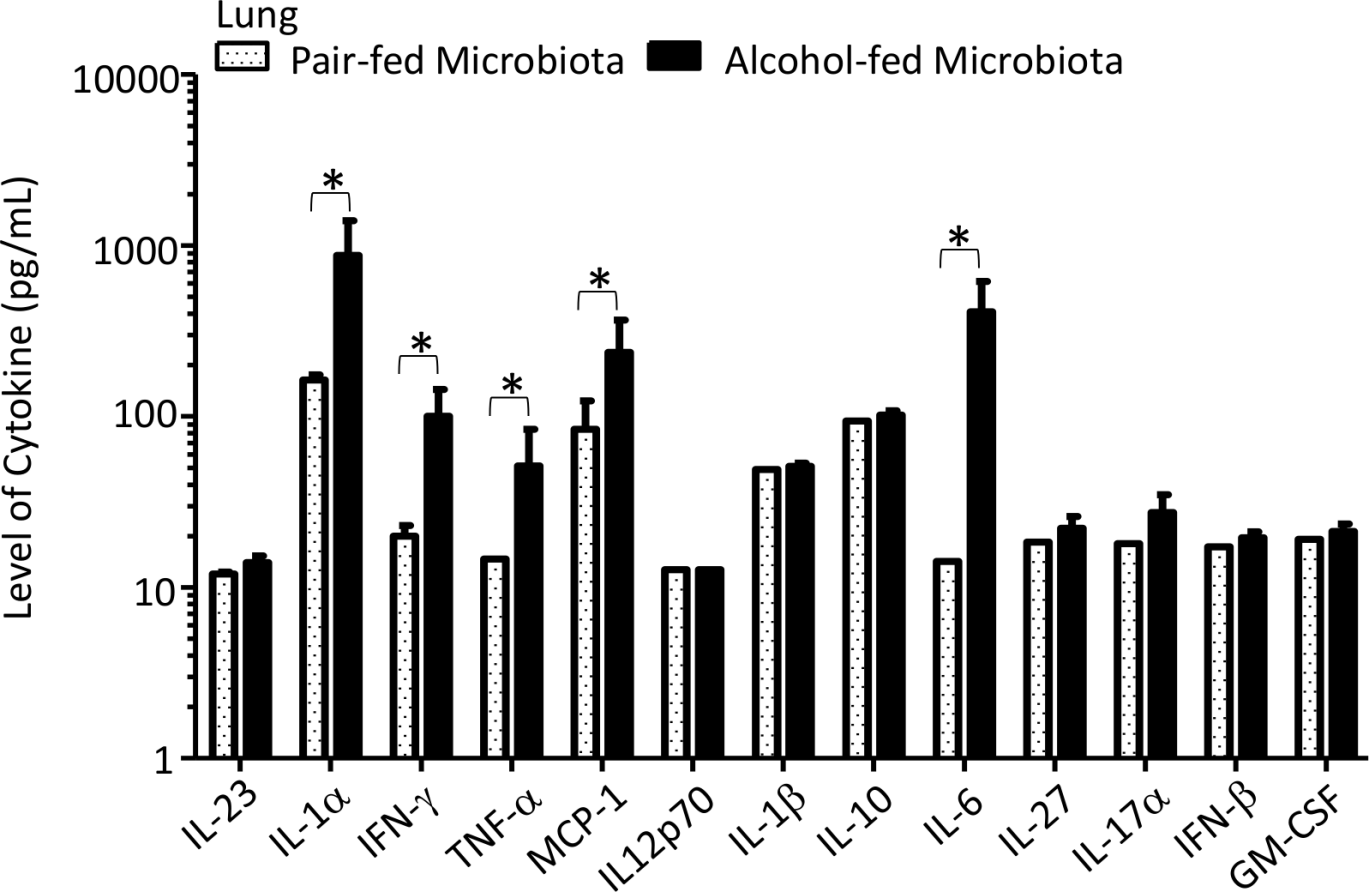
Alcohol-associated dysbiosis alters acute pulmonary inflammatory cytokines. Levels of pulmonary acute inflammatory cytokines were asssesed 48 hrs. post-*Klebsiella* infection via LEGENDplex 13-ples assay kit. Bars represent the mean cytokine level ± SEM, * indicates P < 0.05, by ANOVA with Dunn’s correction. N = 10/group.

We then investigated pulmonary macrophage and dendritic cell populations, as well as acute inflammatory cytokines, following *K*. *pneumoniae* infection. CD103+ dendritic cells (CD11b^+^, CD11c^+^, MHCII^+^, CD103^+^), DC/interstitial macrophages (CD11b^+^, CD11c^+^, MHCII^+^, CD103^-^), and alveolar macrophages (CD11b^-^, CD11c^+^, MHCII^-^, CD103^-^) were significantly different between mice recolonized with microbiota from alcohol-fed mice and pair-fed microbiota recolonized animals following *K*. *pneumoniae* (Fig 11). We also evaluated the levels of pulmonary acute cytokines between alcohol-dysbiosis and pair-fed recolonized mice following *K*. *pneumoniae* infection. We found that the levels of IL-1α, INF-γ, TNF-α, MCP-1, and IL-6 in the lungs post infection were significantly different between mice recolonized with microbiota from alcohol-fed and pair-fed microbiota (Fig 12). Conversely the levels of IL-23, IL12p70, IL-1β, IL-10, IL-27, IL-17α, IFN-β, and GM-CSF were not significantly different between mice recolonized with microbiota from alcohol-fed and pair-fed microbiota (Fig 12).

**Fig 13:**
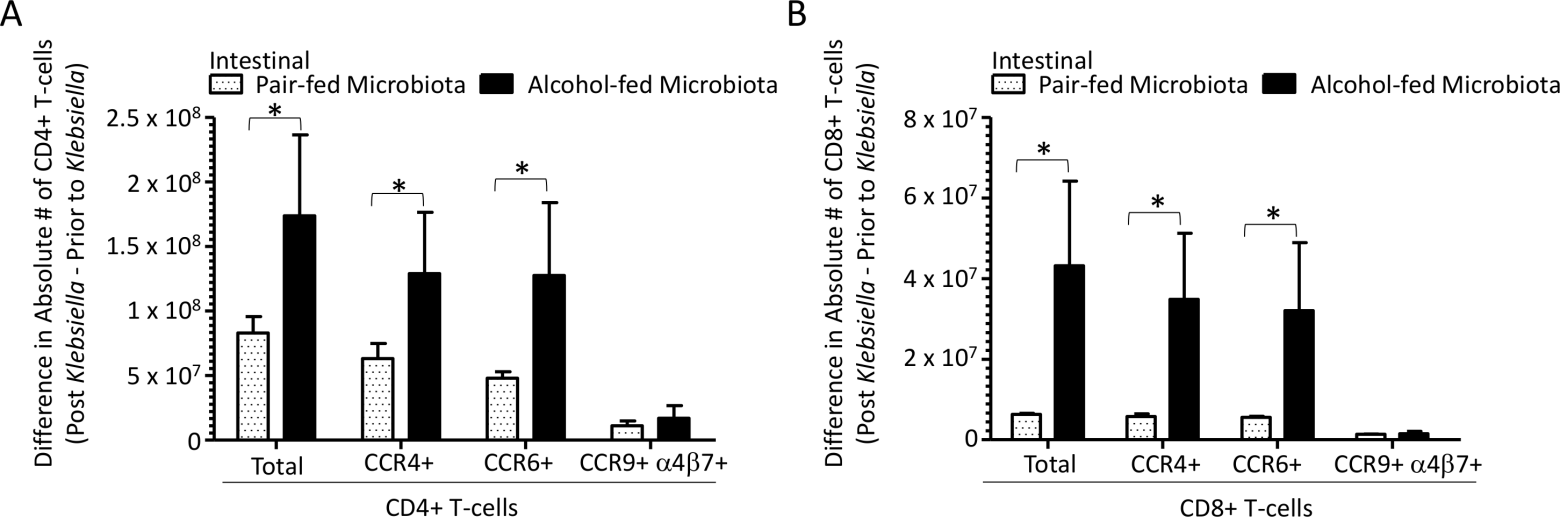
Alcohol-associated dysbiosis increases intestinal T-cell sequestration in mice. (A) Difference in absolute number of homing receptor positive intestinal CD4+ T-cells isolated 48 hrs. post-*Klebsiella* infection from values obtained pre-infection in alcohol-dysbiosis and pair-fed recolonized mice. (B) Difference in absolute number of homing receptor positive intestinal CD8+ T-cells 48 hrs. post-*Klebsiella* infection from values obtained pre-infection in alcohol-dysbiosis and pair-fed recolonized mice. Bars represent the mean of the cell counts post infection minus the cell counts prior to infection ± SEM, * indicates P < 0.05, by ANOVA with Dunn’s correction. N = 10/group.

We sought to characterize the intestinal CD4+ and CD8+ T-cell response, as the microbiota from alcohol-fed mice may be influencing intestinal homeostasis. We found that mice recolonized with an alcohol-associated microbiota had significantly more CD4+ and CD8+ T-cells in the intestinal tract when compared to pair-fed microbiota recolonized mice (Fig 13A and 13B, respectively). In addition, the number of CD4+ and CD8+ T-cells that express the lung homing receptor CCR4, and the dual tropism (gut/lung) homing receptor CCR6 were increased in the intestinal tract of mice recolonized with microbiota from alcohol-fed mice compared to pair-fed microbiota recolonized animals (Fig 13A and 13B, respectively). Moreover, an increase in central memory CD4+ T-cells (S6A Fig) and an increase in the number of intestinal TEMRA CD8+ T-cells was observed in mice recolonized with microbiota from alcohol-fed mice compared to pair-fed microbiota recolonized animals (S6B Fig).

## Discussion

Intestinal bacterial overgrowth is a recognized consequence of excessive alcohol consumption [17, 18]. Chronic alcohol consumption increases intestinal permeability and translocation of bacterial components leading to local and systemic inflammation [34]. The GI tract plays a central role in immune system homeostasis, as it contains 70% of the lymphoid system [35]. It is now evident from multiple lines of investigation that the composition of the intestinal microbiota can directly and/or indirectly influence the hosts’ ability to coordinate and regulate optimal immune response both locally and at distal mucosal sites [36–38]. However, the impact of alcohol-associated dysbiosis on host defense against respiratory infection, independent of the direct effects of alcohol consumption, had not been previously studied. As such, the objective of our study was to investigate the effects of alcohol-associated dysbiosis on pulmonary host defense.

Binge-on-chronic alcohol consumption significantly increased host susceptibility to *Klebsiella* pneumonia, similar to previous reports using different alcohol model systems [10, 13, 14]. Importantly, the increased susceptibility to *Klebsiella pneumoniae* in alcohol-fed animals was, in part, mediated by alcohol-associated intestinal dysbiosis, independent of the direct alcohol effects. This is the first report showing that alcohol-induced dysbiosis is an underlying mechanism for impaired host defense against bacterial infections. Similar observations have been made for various other disease conditions. For example alterations to the immune responses in the gut directly affect the development of allergic disease in the lung, as dysbiotic mice display increased CD4+ T-cell mediated inflammation in the lung following allergen challenge compared to mice with a normal GI flora [39–41]. Furthermore, there is a growing appreciation that gut-derived sepsis induces or amplifies acute respiratory distress syndrome (ARDS) [42]. Specifically, gut-associated bacteria are enriched in the lungs in an experimental sepsis model, as well as in humans with ARDS, suggesting that gut–lung translocation and dysbiosis may contribute to the development of ARDS [21]. Further, the composition of the intestinal microbial communities contributes to pulmonary host defense against bacterial pneumonia, as optimal host defense against *Escherichia coli* pneumonia requires intestinal toll-like receptor signaling [8], and germ-free mice have a strikingly higher mortality rate following *Pseudomonas aeruginosa* pneumonia [9]. Similarly, recently published data demonstrate that the gut microbiota regulates Th17 CD4 T-cell polarization during pulmonary fungal infections [36]. Several additional studies have demonstrated that the gut microbiota promotes pulmonary immunity and resistance to pneumonia [43–46]. Our results suggest that GI dysbiosis may be an underlying mechanism contributing to impaired pulmonary health and increased susceptibility to bacterial infections in chronic alcoholics.

CD4+ and CD8+ T cells expressing the homing receptors CCR4 (lung) and CCR6 (dual tropism gut/lung) were significantly decreased in the lungs of mice recolonized with microbiota from alcohol-fed mice, as well as in binge-on-chronic alcohol-fed mice. This suggests that alcohol-dysbiosis diminishes lung-specific T-cell trafficking impairing lung host defense against *Klebsiella*. Conversely, the number of CD4+ and CD8+ T-cells expressing the homing receptors CCR4 and CCR6 were higher in the intestinal tract of mice recolonized with microbiota from alcohol-fed mice. These results suggest that CD4+ and CD8+ T-cells programmed to home to the lung are sequestered in the intestine, impairing lung host defense against *Klebsiella*. To confirm the importance of T-cells in the defense against *Klebsiella* pneumonia we depleted CD4+ and CD8+ T-cells prior to respiratory infection with *Klebsiella pneumoniae*. Our results show that both CD4+ and CD8+ T-cells are required for maximal clearance of *K*. *pneumoniae*. This is consistent with previous reports showing that secretion of IFN-γ from Th1 cells is required for maximal clearance of *Klebsiella* [14], that both CD4+ and CD8+ T-cell are required for maximal host defense and survival following *Klebsiella* septicemia [47], and that disruption of the IL-23 axis increases susceptibility to *Klebsiella* infection [10]. Our data suggest that alcohol impairs T-cell responses and may explain the propensity for *K*. *pneumoniae* in AUDs. However, additional studies are need to fully understand the role of T-cell in host defense against *Klebsiella pneumoniae* during excessive alcohol use, as well as to define the role of the intestinal microbiota in shaping the T-cell response.

Similarly, macrophages and dendritic cells were significantly decreased in the lungs of mice recolonized with microbiota from alcohol-fed mice, as well as in binge-on-chronic alcohol-fed mice, while the levels of acute inflammatory cytokines (i.e., IL-1α, INF-γ, TNF-α, MCP-1, and IL-6) were significantly increased in the lungs of mice recolonized with microbiota from alcohol-fed mice, as well as in binge-on-chronic alcohol-fed mice. Increased pulmonary cytokine levels in mice recolonized with microbiota from alcohol-fed mice, as well as in binge-on-chronic alcohol-fed mice in the absence of increased innate and adaptive immune cells may occur through several potential mechanisms; (a) increased pulmonary cytokine levels are driven by systemic inflammation (b) increased bacterial burden drives increased inflammation independent of immune cell recruitment, and/or (c) immune cells in the lung are hyper-responsive in alcohol-dysbiosis or binge-on-chronic alcohol-fed mice. These data suggests that alcohol-dysbiosis diminishes the pulmonary macrophage and dendritic cell response, while increasing acute pulmonary inflammation. These results are similar to previous reports demonstrating that increased pulmonary inflammation occurs following burn treatment in alcohol consuming animals via liver mediated secretion of IL-6 [48]. Taken together, these results suggest that increased pulmonary inflammation without immune cell recruitment (i.e., macrophages and T-cells) may lead to inflammation and/or damage of the lung (minus the protective and/or phagocytic immune cells) thus enabling *Klebsiella pneumoniae* growth and infection.

Mice recolonized with an alcohol-dysbiotic microbiota also exhibited increased levels of biomarkers of intestinal barrier damage. These results are similar to those previously observed, which demonstrate that conventionalization of germ-free mice with intestinal contents from alcohol-fed conventional mice induces inflammation in the small intestine [23]. Additionally, increased intestinal damage/permeability following an alcohol/burn treatment was associated with increased pulmonary inflammation, and restoration of gut barrier function resulted in decreased pulmonary neutrophil infiltration and decreased alveolar wall thickening [48]. These results suggest that alterations to intestinal damage and/or permeability may influence pulmonary host defense by increase pulmonary inflammation and damage. However, very little is known regarding the role of intestinal barrier damage in host defense against respiratory pathogens, or how alcohol-dysbiotic microbiota may participate in these processes.

We investigated the consequences of binge-on-chronic alcohol consumption on the intestinal microbial communities and metabolic constituents. Binge-on-chronic alcohol consumption in mice lead to intestinal dysbiosis characterized by an increase in the relative abundance of the Gram-negative organisms *Bacteroides acidifaciens*, *Bacteroides eggerthii* and *Oscillospria spp*. and a decrease in *Bacteroides uniformis*, *Parabacteriodes gordonii*, and *Akkermansia muciniphilla*. These results are similar to previously published results, which found that ethanol feeding reduced the phylum *Firmicutes* [21, 22] and the genus *Lactobacillus* [20, 21], while *Enterococcus* [21, 23], *Akkermansia*, *Corynebacterium*, and *Alcaligenes* spp. increased after alcohol administration [20–22]. We did observe several differences compared to other published results (i.e., a decrease in *Akkermansia* v. an increased abundance). There are several possible explanations for these contradictive findings including: (1) differences in the alcohol diet model used (binge-on-chronic v. intragastric feeding), and/or (2) duration of the alcohol feeding protocol (1-wk v. 3-wks) [21].

Functional changes associated with intestinal dysbiosis were characterized using a metabolomics approach. Using Ultra Performance Liquid Chromatography-High Resolution Mass Spectrometry (UPLC-HRMS), a Dionex Ultimate 3000 was coupled to a Thermo Scientific Exactive Plus Orbitrap to evaluate the microbial metabolic changes associated with binge-on-chronic alcohol feeding, it was found that binge-on-chronic alcohol consumption significantly increased the concentrations of the metabolite allantoin, and in contrast significantly decreased the concentrations of aconitate, kynurenic acid, d-gluconate, and xanthurenic acid. Previously, chronic alcohol consumption has been associated with marked reduction in amino acid metabolism, perturbations of steroid, lipid, carnitine [24], and bile acid metabolism [25]. These results are consistent with those reports as two metabolic products of amino acid (tryptophan) catabolism (i.e., kynurenic acid and xanthurenic acid) were significantly decreased following binge-on-chronic alcohol consumption. Additionally, aconitate, a tricarboxylic acid cycle intermediate, was decreased following binge-on-chronic alcohol feeding, suggesting ethanol-mediated inhibition of the tricarboxylic acid cycle [49].

These changes are particularly relevant to our findings as all of the bacterial species, as well as, the bacterial metabolites mentioned have been associated with altered host hemostasis, metabolism, and/or immunity. *Bacteroides* species are significant clinical pathogens, participate in the maintenance of a complex and beneficial relationship intestinal homeostasis, and can influence the host immune system so that it controls competing pathogens [50]. Similarly, *Lactobacillus spp*. have been shown to attenuated infection with *Salmonella* Typhimurium, as well as conferred resistance to pulmonary infection with *Streptococcus pneumoniae* [51]. *Lactobacillus* has also been shown to mitigate pulmonary infection with *Pseudomonas aeruginosa* [52]. *Akkermansia* has been linked with intestinal health and improved metabolic status, through a reduction in high fat diet-induced endotoxemia, which developed as a result of an impaired gut barrier [53, 54]. Additionally, *Akkermansia* treatment has been demonstrated to improved enterocyte monolayer integrity [55], suggesting that *Akkermansia spp*. may play a critical role in intestinal barrier maintenance and regulation.

Allantoin, a biomarker used to reflect oxidative stress, is increased during chronic illnesses and participates in immune senescence [56]. Aconitate is metabolized to itaconic acid, which is a potent inhibitor of bacterial growth [57]. Ca-gluconate has been shown to reduce expression of pro-inflammatory cytokines IL-6 and TNF-α, as well as myeloperoxidase levels in both burn and rheumatoid arthritis mouse models [58]. Further, the tryptophan catabolites (TRYCATs), kynurenine, kynurenic acid, xanthurenic acid, and quinolinic acid maintain and regulate the indoleamine 2,3-dioxygenase (IDO) pathway [59]. IDO is an important immunomodulatory enzyme produced by some alternatively activated macrophages, as well as other immune regulatory cells. Furthermore, LPS-induced secretion of IFNγ was shown to be significantly decreased following treatment with TRYCATs [59]. Kynurenine, kynurenic acid, and xanthurenic acid, also decreased the IFNγ/IL-10 ratio, while only kynurenic acid significantly reduces the concentration of TNFα following LPS treatment [59]. Taken together, our results, combined with previously published findings, suggest that the functional (metabolomics) changes to the intestinal microbial communities during harmful alcohol-use may represent an underlying mechanism by which alcohol alters host defense and inflammation. In addition, our results lay the foundation for future studies investigating the potential microbial species and/or microbial metabolites with are immunoprotective and immunosuppressive.

The intestinal microbiota exhibits dynamic responses to external stimulation (i.e., alcohol). In addition, small changes to this community over time may act differently than drastic changes over a short time, making it difficult to model dysbiosis temporally. However, we feel that our model system includes time as an aspect in the modeling of microbiota differences, as the animals were exposed to alcohol for 13 days (11 without and 2 with infection), while the adoptive transfer animals were exposed to the microbial communities for 12 days (10 without and 2 with infection) thus the time of microbial community interaction with the host is similar. It is also plausible that in the setting of continuous alcohol consumption that interactions between the microbiota, alcohol, and the host immune system occur, suggesting that chronic alcohol consumption and intestinal dysbiosis may act in a two-hit model paradigm thus increasing host susceptibility to pneumonia. Likewise it is important to recognize that diet is an important aspect of microbial alterations, as well as a potential factor contributing to alcohol-mediated immune regulation. Therefore future studies are needed to evaluate the effects of different diets, as well as different alcohol consumption model paradigm to fully clarify the role of alcohol-associated intestinal dysbiosis on pulmonary host defense.

## Summary

**Fig 14:**
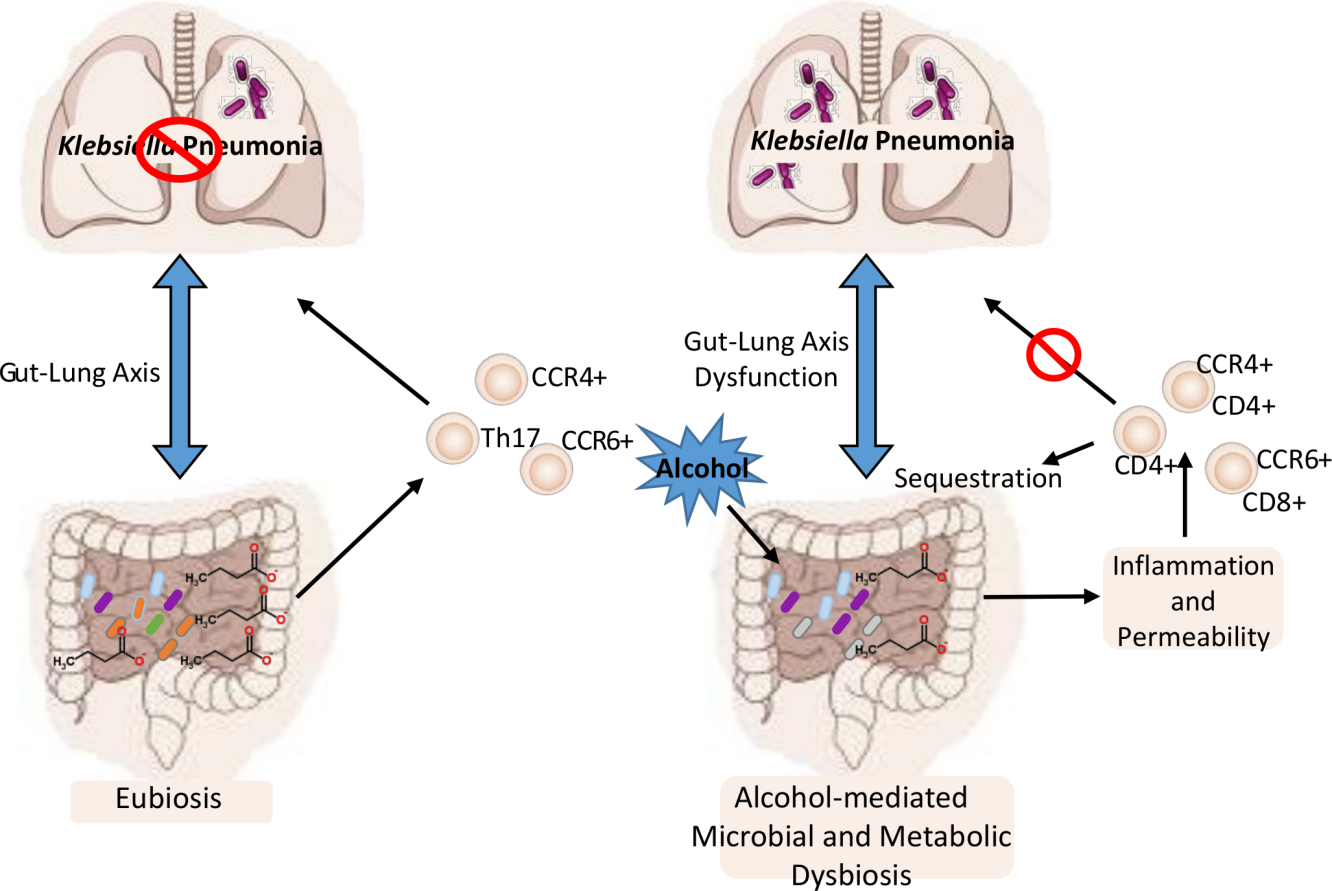
Working model for the effects of alcohol-dysbiosis on host defense against pulmonary pathogens. Alcohol promotes intestinal microbial and metabolic dysbiosis, which increases intestinal inflammation and permeability leading to intestinal T-cell sequestration and impaired T-cell trafficking to the respiratory tract, all of which, in combination, increase host susceptibility to respiratory infection with *Klebseilla pneumoniae*.

The results from these studies show that increased susceptibility to *Klebsiella pneumoniae* in alcohol-fed animals is, in part, mediated by gut dysbiosis, as alcohol-naïve animals recolonized with a microbiota isolated from alcohol-fed mice had increased susceptibility to *K*. *pneumoniae* compared to mice recolonized with a control microbiota. The increased susceptibility in alcohol-dysbiosis recolonized animals was associated with a decrease number of CD4+ and CD8+ T-cells in the lung and increased numbers of T-cells in the intestinal tract following *Klebsiella* infection, suggesting that intestinal sequestration of T-cells may contribute to impaired host defense from lung infections. Mice recolonized with an alcohol-dysbiotic microbiota also exhibited increased pulmonary inflammation and increased biomarkers of intestinal barrier damage, consistent with a mechanistic role for intestinal permeability and chronic inflammation in modulating the enhanced susceptibility to *Klebsiella pneumoniae* during binge-on-chronic alcohol consumption. Fig 14 shows our working model for how alcohol-dysbiosis increases susceptibility to *Klebsiella* pneumonia. These data also expand our current understanding of AUD-associated pneumonia and may, in the future, lead to new preventative and/or therapeutic approaches.

## Methods

### Ethics statement

All experiments were approved by the Louisiana State University Health Sciences Center (LSUHSC) Institutional Animal Care and Use Committee, protocol number 3358. LSUHSC utilizes the Public Health Service Policy on Humane Care and Use of Laboratory Animals (PHS) and uses the Guide for the Care and Use of Laboratory Animals as a basis for establishing and maintaining an institutional program for activities involving animals. LSUHSC animal care policies comply with all applicable provisions of the Animal Welfare Act (AWAR), guidance from the Office of Laboratory Animal Welfare (OLAW), the American Veterinary Medical Association Guidelines on Euthanasia, and all state and local regulations.

### Mice

Male 8 to 10 week old C57BL/6 mice were obtained from Charles Rivers Breeding Laboratories (Wilmington, MA) and maintained in a temperature (72^°^C) controlled room for two days prior to experimental manipulation. Animals were housed in filter-topped cages and were provided autoclaved water and chow *ad libitum*. Animals were kept in the animal care facility at LSUHSC throughout the experiment. Animals were handled under a laminar flow hood to maintain SPF conditions throughout the course of the experiment.

### Klebsiella pneumoniae infection

*Klebsiella pneumoniae* infections were performed as previously described [10, 13]. Briefly, *K*. *pneumoniae* (strain 43816, serotype 2; American Type Culture Collection, Manassas, VA) were grown in 100 mL tryptic soy broth (Becton Dickinson, Franklin Lakes, NJ) in a shaking incubator (185 r.p.m.) at 37 ^o^C for 18 hours. Bacteria were then pelleted by centrifugation (2,000 x g for 15 minutes at 4 ^o^C), washed twice with phosphate-buffered saline (PBS), and resuspended in PBS at an estimated concentration of 1 x 10^3^ colony-forming units (CFU)/mL. The actual number of viable bacteria was determined by serial dilutions onto HiCrome *Klebsiella* Selective Agar plates and performing standard colony counts. Twenty four hours after the final ethanol binge, animals were anesthetized with isoflurane and given 1 x 10^2^ CFU bacteria in 100 μL PBS via intratracheal (IT) administration using a P200 pipette. Animals were allowed to recover from anesthesia and returned to their cages. Mice were sacrificed 48 hrs. post infection. This time point was selected because it allows assessment of pathogen burden and host defense, without substantial mortality.

### Binge-on-chronic alcohol model

We have adapted and modified the NIAAA chronic-binge alcohol model [33] to generate alcohol-dysbiotic microbiota and pair-fed microbiota for adoptive transfer. Briefly, 8 to 10-week-old male C57BL/6 mice were acclimated to liquid diet for 5 days using Lieber-DeCarli ‘82 Shake and Pour control liquid diet (Bioserv, Flemington, NJ). Groups of mice (n = 1–2 per cage) were randomized into ethanol fed (Lieber-DeCarli ‘82 Shake and Pour 5% vol/vol ethanol liquid diet) or pair-fed groups (control-liquid diet). Pair-fed mice were maintained on control-liquid diet adjusted daily according to the consumption of ethanol-fed mice. Mice were then administered 4 g kg^-1^ (24.03% vol/vol) ethanol by gavage (binge) following 5 days of chronic-ethanol consumption. Pair-fed control mice were gavaged with 9 g kg^-1^ (45% wt/vol) maltose dextrin. Mice were maintained on the 5% ethanol diet for an additional 5 day period and on day 10, mice received a second and final ethanol binge (4 g kg^-1^). Mice were sacrificed 24 hours following the last binge ethanol administration and the cecal microbiota was collected, homogenized, diluted, and stored at -80^°^C for use in the fecal transfer experiments. A similar cohort of mice were infected with 1 x 10^2^ CFU of *K*. *pneumoniae* via i.t. inoculation and sacrificed 48 hrs. post infection.

### Microbiota collection and fecal adoptive transfer

Microbiota adoptive transfer was performed as previously described [60]. Briefly, alcohol-naïve 8 to 10-week-old male control liquid diet fed C57BL/6 mice were treated (oral gavage) with a cocktail of antibiotics (ampicillin, gentamicin, neomycin, metronidazole [all at 0.25 mg/day], and vancomycin [0.125 mg/day]) daily for two weeks. Cecal content collected from alcohol-fed or pair-fed mice (see above) was homogenized and prepared for recolonization. Cecal content was weighed and homogenized (1:2 wt/vol) in sterile PBS. Samples were then vigorously mixed and placed on ice for 10 min to allow organic matter to settle out. Supernatants were collected and passed through sterile 2-ply gauze to remove any large organic material and either used immediately or stored at -80^°^C. Microbiota-depleted mice were then recolonized with 200 μl of the cecal microbiota from either alcohol-fed or pair-fed mice donors, by gavage on days 2 and 5 post-antibiotic treatment. One week following the final microbiota recolonization mice were sacrificed and baseline immune and physiological characteristics were determined. One week recolonization is sufficient to induce immune modulation and homeostasis in germ-free mice [61]. A similar cohort of mice were infected with 1x10^2^ CFU of *K*. *pneumoniae* via i.t. inoculation and sacrificed 48 hrs. post infection.

### DNA sequencing of the 16s rRNA gene

Sequencing and bioinformatics analysis were performed by the Louisiana State University School of Medicine Microbial Genomics Resource Group (http://metagenomics.lsuhsc.edu/mgrg), as we have previously published [62, 63].

### Sequence curation and analysis

16S rRNA gene sequences were curated using Quantitative Insights Into Microbial Ecology (QIIME 1.9.1) and the R package Phyloseq scripts, as described previously [60, 62, 64].

### Metabolomics analysis

Metabolic profiles of binge-on-chronic alcohol and pair-fed mice were determined by Ultra Performance Liquid Chromatography-High Resolution Mass Spectrometry (UPLC-HRMS), via a Dionex Ultimate 3000 coupled with a Thermo Scientific Exactive Plus Orbitrap, as described previously [65]. Raw metabolomics data files generated by Xcalibur were converted to open-source mzML format [66] using ProteoWizard [67]. Non-linear retention time correction were computed by MAVEN [68] for each sample and metabolites were identified using retention time and exact mass within 5 ppm window. Relative concentrations were normalized using mass of processed samples and heat maps were generated by a log_2_ fold change using Cluster [69] and Java Treeview [70]. Partial least squares discriminant analysis (PLSDA) score plots were made with the DiscriMiner package within R [71].

### Flow cytometric analysis of lymphocytes from lung and intestinal tissue

Lung and intestinal tissue of each animal was minced; suspended in 10 ml homogenization buffer consisting of RPMI 1640 with 1 mg/ml Collagenase type 1 (Worthington Biochemical, Lakewood, NJ) and 30 μg/ml DNase I (Roche Diagnostics, Indianapolis, IN); and incubated at 37°C with shaking for 30 min. Cell suspensions were further disrupted by passing through a 70-μm nylon mesh. Intestinal lymphocytes were further purified by 44%/67% Percoll gradient [72]. Red blood cells were lysed using RBC lysis buffer (BioLegend, San Diego, CA) prior to staining. After washing with PBS, viable cells were counted on a hemocytometer using the trypan blue–exclusion method. One million viable cells were stained with the LIVE/DEAD Fixable Dead Cell Stain Kit (Invitrogen Eugene, OR) followed by immunological staining with various combinations of fluorochrome-conjugated Abs specific for murine CD45, CD3e, CD4, CD8a, CD44, CD62L, CCR6, CCR4, CCR9, CXCR3, α4β7, CD45RA, and CCR7 (BioLegend) suspended in FACS buffer at pre-determined concentrations for 30 min at 4°C. All cells were pretreated with TruStain FcX Anti-mouse CD16/32 antibody (BioLegend). Wells were then washed with FACS buffer and fixed with PBS + 1% formalin. For all experiments, cells were acquired using an LSR II flow cytometer (BD Biosciences, San Jose, CA), and analyses were performed using FlowJo software Version 9.4 (Tree Star, Ashland, OR).

### Serum EtOH, Alanine Aminotransferase (ALT), and intestinal fatty acid binding protein (i-FABP analysis

Whole blood was either collected from tail bleeds or cardiac puncture, and serum was obtained via centrifugation of whole blood in BD serum separator tubes at 1,500 x g for 10 min. at 4^°^C. Serum was stored at -20 ^o^C for later use. Blood alcohol content was determined using the Ethanol Assay Kit (Sigma-Aldrich, St. Louis, MO.) according to manufacturer’s instructions. Circulating alanine aminotransferase levels were determined by ELISA (Cloud-Clone Corp., Houston, TX.) following recommended instructions. Levels of i-FABP were assessed via ELISA (LifeSpan BioSciences, Seattle, WA.) according to manufactures’ instructions.

### Mouse inflammation panel

Lung tissue of each animal were minced and suspended in 1 ml homogenization buffer and further disrupted by passing through a 70-μm nylon mesh. Cells and debris were removed from the suspension via centrifugation at 300 x g for 10 min and supernatant were collected and stored at -80^°^C for later use. Lung cytokine levels were assessed from the supernatant samples via LEGENDplex (mouse inflammation panel 13-plex; BioLegend) kit per manufacturer’s instructions. Cytokine levels were acquired using a FACSCanto II flow cytometer (BD Biosciences, San Jose, CA), and analyses were performed using LEGENDplex data analysis software (BioLegend).

### CD4 and CD8 T-cell depletion

Mice were depleted of CD4+ or CD8+ T-cells by intraperitoneal (i.p.) injection of 100 μg anti-CD4+ mAb (hybridoma GK1.5; National Cell Culture Center) and 100 μg of anti-CD8 mAb (hybridoma 58.6.72; National Cell Culture Center) in 100 μl PBS 3 days prior to infection, respectively. This treatment protocol results in >97% sustained depletion of CD4+ and CD8+ lymphocytes from blood and lymphoid tissue for up to 14 wk [73, 74].

### Statistical analysis

Results are presented as mean ± S.E.M. Statistical analyses were performed using GraphPad Prism 5 (La Jolla, CA. USA) and statistical significance was measured at P ≤ 0.05. For comparisons between two groups a Mann-Whitney U with both Benjamini-Hochberg and Bonferroni corrections for multiple comparisons was performed. A 1-way ANOVA followed by a Kruskal-Wallis test was performed for groups of 3 or more.

## Supporting information

S1 Fig Volume consumed during alcohol feeding. Levels of liquid diet consumed were measured daily after the start of alcohol feeding. Bars represent the mean volume of diet consumed in alcohol-fed and pair-fed cages.
(TIFF)

S2 Fig Binge-on-chronic alcohol use alters the intestinal microbial communities. Alcohol-treated mice (red circles) showed significantly different microbial community structures from pair-fed mice (blue circles) as determined by principal coordinate analysis of the unweighted UniFrac metric via Qiime.
(TIFF)

S3 Fig Binge-on-chronic alcohol use decreases the number of lung CD4+ and CD8+ T-cells in the lung following *Klebsiella* infection. (A) The difference in absolute number of lung Th1, Th2, Th17, CM and EM CD4+ T-cells 48 hrs. post-*Klebsiella* infection from alcohol-fed and pair-fed mice. (B) The difference in absolute number of lung CM, EM, and TEMRA CD8+ T-cells 48 hrs. post-*Klebsiella* infection from alcohol-fed and pair-fed mice. Bars represent the mean of the cell counts post infection minus the cell counts prior to infection ± SEM. * indicates P < 0.05, by Mann-Whitney U or by ANOVA with Dunn’s correction. N = 10/group.
(TIFF)

S4 Fig CD4+ and CD8+ T-cells are required for maximal host defense against *Klebsiella pneumoniae*. (A) *Klebsiella* lung burden at 48 hrs. post infection in control (isotype), as well as CD4+ and CD8+ T-cell depleted mice. (B) The percent of lung CD4+ and CD8+ T-cells following monoclonal antibody depletion prior to respiratory tract infection. * indicates P < 0.05, by Mann-Whitney U. N = 10/group.
(TIFF)

S5 Fig Alcohol-associated dysbiosis decreases the number of lung CD4+ and CD8+ T-cells in the lung following *Klebsiella* infection. (A) The difference in absolute number of lung Th1, Th2, Th17, CM and EM CD4+ T-cells isolated 48 hrs. post-*Klebsiella* infection from mice recolonized with the intestinal microbiota from alcohol- or pair-fed mice. (B) The difference in absolute number of lung CM, EM, and TEMRA CD8+ T-cells 48 hrs. post-*Klebsiella* infection from mice recolonized with the intestinal microbiota from alcohol- or pair-fed mice. Bars represent the mean of the cell counts post infection minus the cell counts prior to infection ± SEM. * indicates P < 0.05, by ANOVA with Dunn’s correction. N = 10/group.
(TIFF)

S6 Fig Alcohol-associated dysbiosis increases the number of intestinal CD4+ and CD8+ T-cells in the lung following *Klebsiella* infection. (A) The difference in absolute number of intestinal Th1, Th2, Th17, CM and EM CD4+ T-cells isolated from mice recolonized with the intestinal microbiota from alcohol- or pair-fed mice. (B) The difference in absolute number of lung CM, EM, and TEMRA CD8+ T-cells isolated from mice recolonized with the intestinal microbiota from alcohol- or pair-fed mice. Bars represent the mean of the cell counts post infection minus the cell counts prior to infection ± SEM. * indicates P < 0.05, by ANOVA with Dunn’s correction. N = 10/group.
(TIFF)
