# Supplementary figures and images for: Alcohol-associated intestinal dysbiosis impairs pulmonary host defense against Klebsiella pneumoniae
Source: PLoS Pathog. 2017 Jun 12;13(6):e1006426. doi: 10.1371/journal.ppat.1006426 (PMC5481032; doi:10.1371/journal.ppat.1006426)

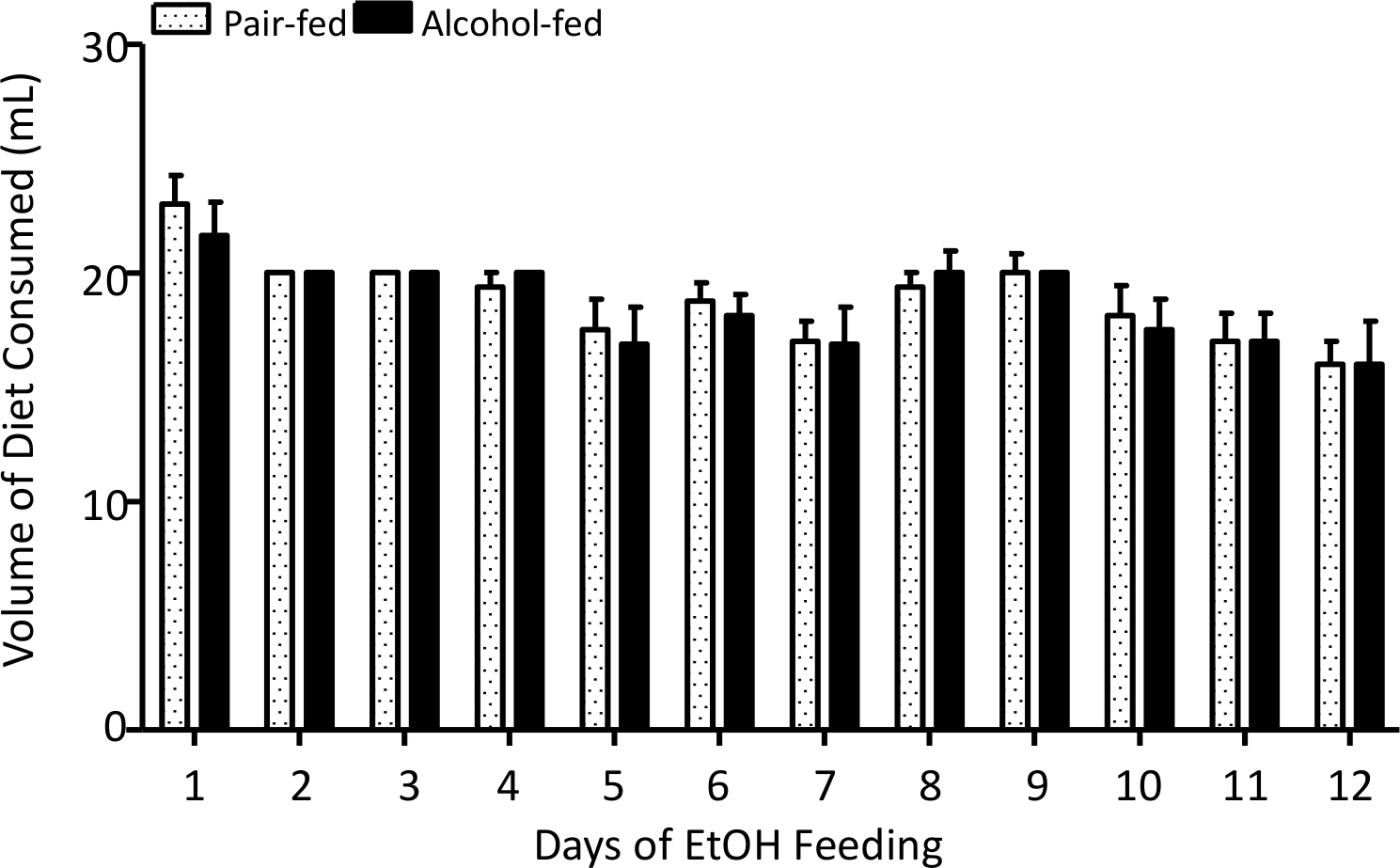

Supplement: S1 Fig — Levels of liquid diet consumed were measured daily after the start of alcohol feeding. Bars represent the mean volume of diet consumed in alcohol-fed and pair-fed cages. (TIFF) [file ppat.1006426.s001.tiff]

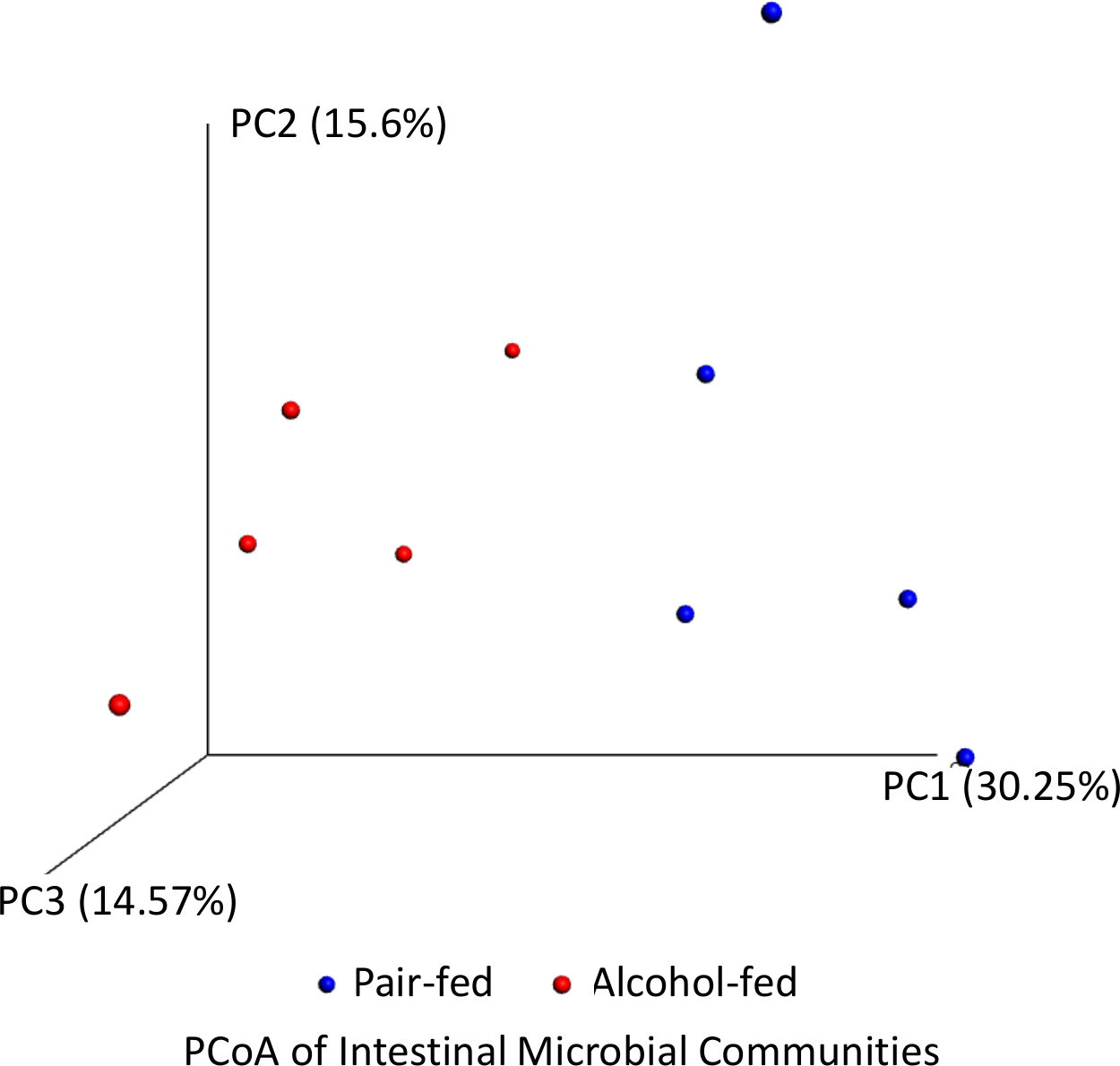

Supplement: S2 Fig — Alcohol-treated mice (red circles) showed significantly different microbial community structures from pair-fed mice (blue circles) as determined by principal coordinate analysis of the unweighted UniFrac metric via Qiime. (TIFF) [file ppat.1006426.s002.tiff]

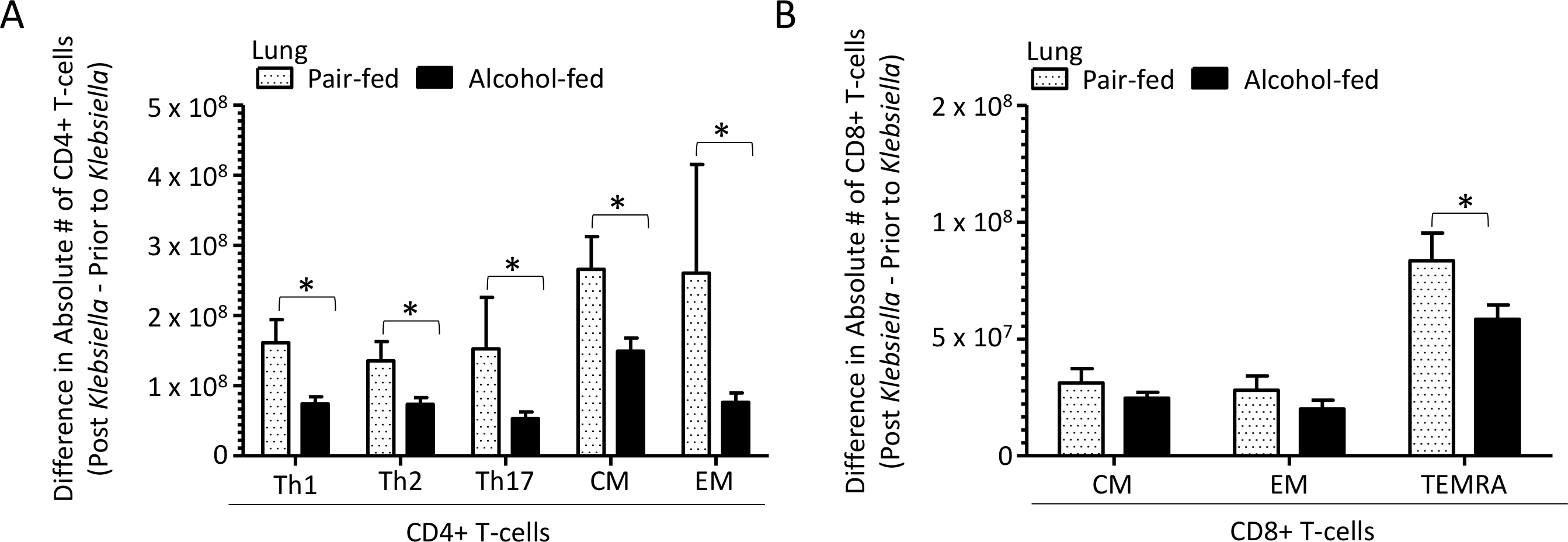

Supplement: S3 Fig — (A) The difference in absolute number of lung Th1, Th2, Th17, CM and EM CD4+ T-cells 48 hrs. post-Klebsiella infection from alcohol-fed and pair-fed mice. (B) The difference in absolute number of lung CM, EM, and TEMRA CD8+ T-cells 48 hrs. post-Klebsiella infection from alcohol-fed and pair-fed mice. Bars represent the mean of the cell counts post infection minus the cell counts prior to infection ± SEM. * indicates P < 0.05, by Mann-Whitney U or by ANOVA with Dunn’s correction. N = 10/group. (TIFF) [file ppat.1006426.s003.tiff]

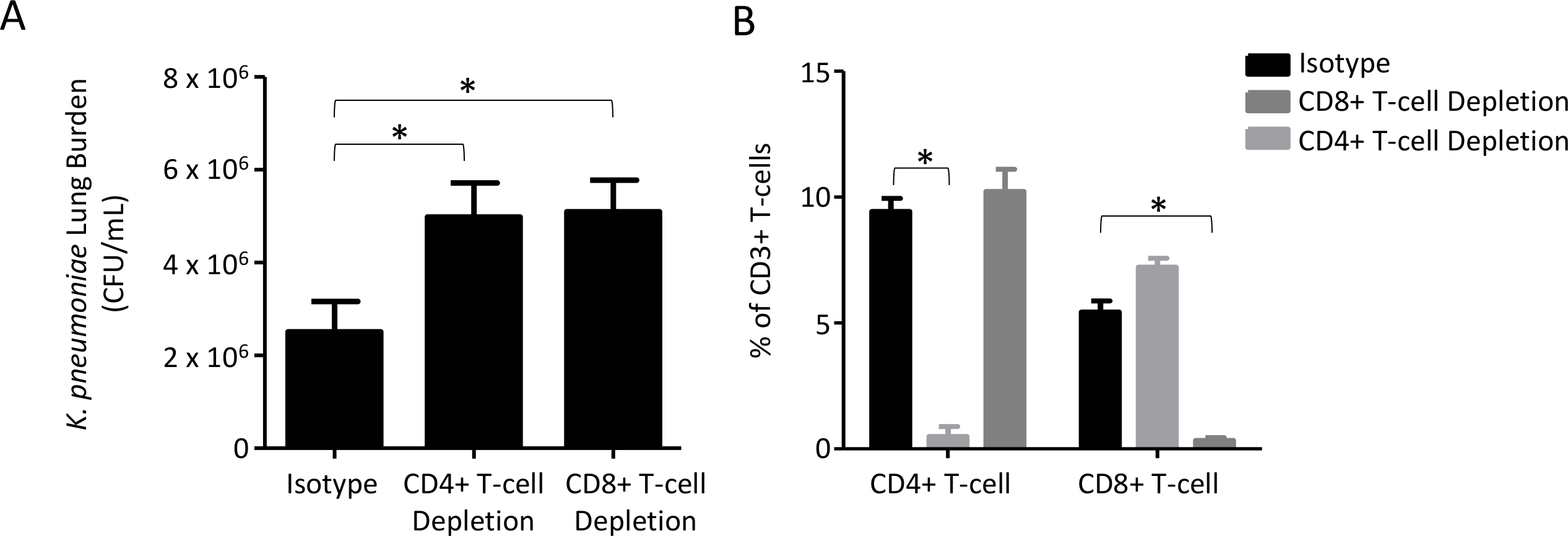

Supplement: S4 Fig — (A) Klebsiella lung burden at 48 hrs. post infection in control (isotype), as well as CD4+ and CD8+ T-cell depleted mice. (B) The percent of lung CD4+ and CD8+ T-cells following monoclonal antibody depletion prior to respiratory tract infection. * indicates P < 0.05, by Mann-Whitney U. N = 10/group. (TIFF) [file ppat.1006426.s004.tiff]

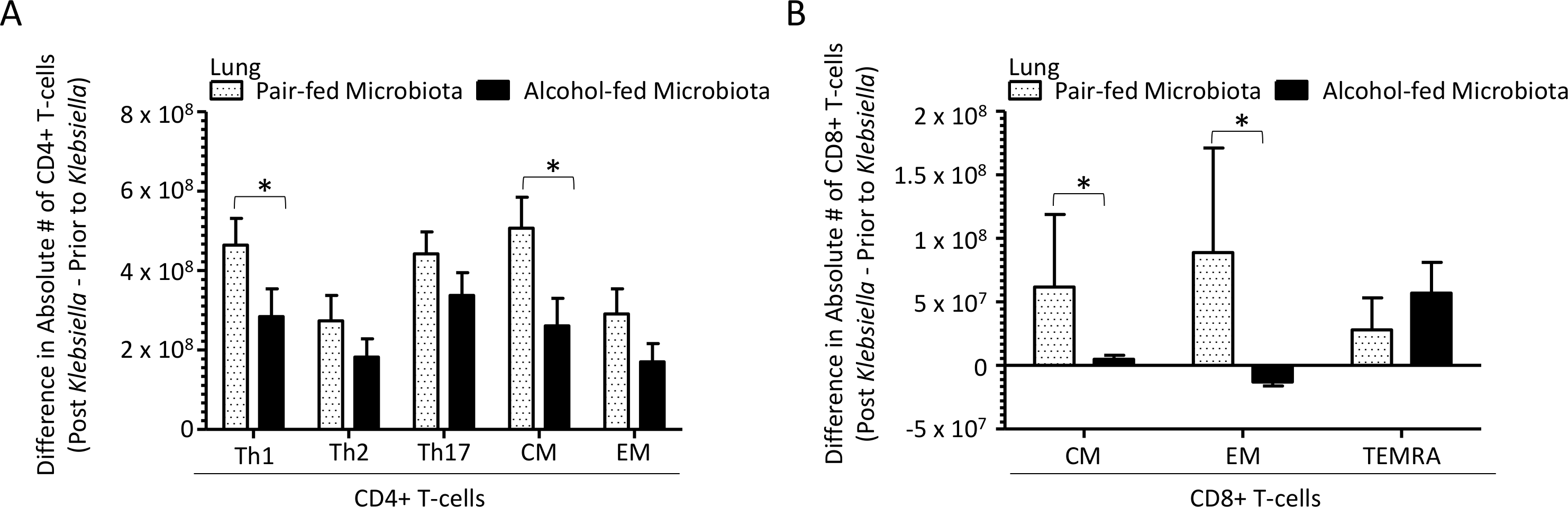

Supplement: S5 Fig — (A) The difference in absolute number of lung Th1, Th2, Th17, CM and EM CD4+ T-cells isolated 48 hrs. post-Klebsiella infection from mice recolonized with the intestinal microbiota from alcohol- or pair-fed mice. (B) The difference in absolute number of lung CM, EM, and TEMRA CD8+ T-cells 48 hrs. post-Klebsiella infection from mice recolonized with the intestinal microbiota from alcohol- or pair-fed mice. Bars represent the mean of the cell counts post infection minus the cell counts prior to infection ± SEM. * indicates P < 0.05, by ANOVA with Dunn’s correction. N = 10/group. (TIFF) [file ppat.1006426.s005.tiff]

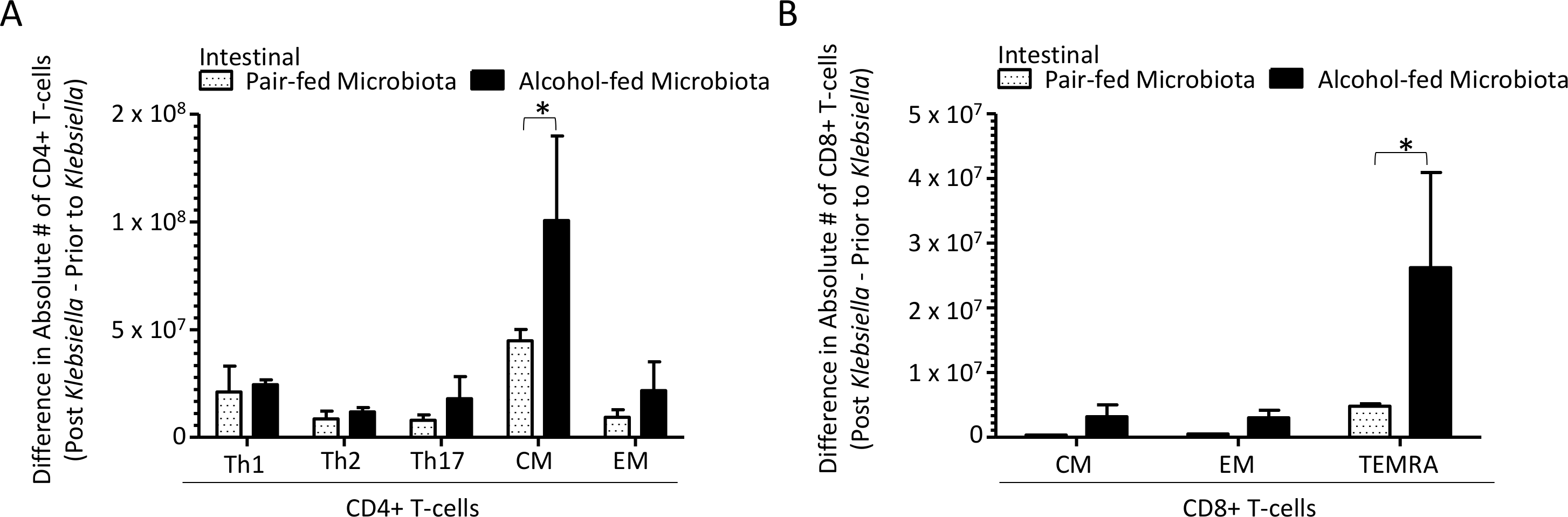

Supplement: S6 Fig — (A) The difference in absolute number of intestinal Th1, Th2, Th17, CM and EM CD4+ T-cells isolated from mice recolonized with the intestinal microbiota from alcohol- or pair-fed mice. (B) The difference in absolute number of lung CM, EM, and TEMRA CD8+ T-cells isolated from mice recolonized with the intestinal microbiota from alcohol- or pair-fed mice. Bars represent the mean of the cell counts post infection minus the cell counts prior to infection ± SEM. * indicates P < 0.05, by ANOVA with Dunn’s correction. N = 10/group. (TIFF) [file ppat.1006426.s006.tiff]
